# Unravelling Protein-Protein Interaction Networks Linked to Aliphatic and Indole Glucosinolate Biosynthetic Pathways in Arabidopsis

**DOI:** 10.3389/fpls.2017.02028

**Published:** 2017-11-29

**Authors:** Sebastian J. Nintemann, Daniel Vik, Julia Svozil, Michael Bak, Katja Baerenfaller, Meike Burow, Barbara A. Halkier

**Affiliations:** ^1^Department of Plant and Environmental Sciences, Faculty of Science, DynaMo Center, University of Copenhagen, Frederiksberg, Denmark; ^2^Department of Biology, ETH Zurich, Zurich, Switzerland

**Keywords:** *Arabidopsis thaliana*, protein-protein interactions, glucosinolates, regulatory networks, pathway organization

## Abstract

Within the cell, biosynthetic pathways are embedded in protein-protein interaction networks. In Arabidopsis, the biosynthetic pathways of aliphatic and indole glucosinolate defense compounds are well-characterized. However, little is known about the spatial orchestration of these enzymes and their interplay with the cellular environment. To address these aspects, we applied two complementary, untargeted approaches—split-ubiquitin yeast 2-hybrid and co-immunoprecipitation screens—to identify proteins interacting with CYP83A1 and CYP83B1, two homologous enzymes specific for aliphatic and indole glucosinolate biosynthesis, respectively. Our analyses reveal distinct functional networks with substantial interconnection among the identified interactors for both pathway-specific markers, and add to our knowledge about how biochemical pathways are connected to cellular processes. Specifically, a group of protein interactors involved in cell death and the hypersensitive response provides a potential link between the glucosinolate defense compounds and defense against biotrophic pathogens, mediated by protein-protein interactions.

## Introduction

The cell is a busy place where many biological processes are regulated by physical or functional protein-protein interaction networks. Within metabolism, many biosynthetic pathways are proposed to be assembled into multi-enzyme complexes called metabolons (Srere, 1985). Organization into metabolons provides several advantages such as increase in local concentrations of the enzymes and their substrates, efficient sequestration of reactive intermediates and improved channeling of intermediates to increase flux (Bassard et al., 2012; Laursen et al., 2015, 2016; Dastmalchi et al., 2016; French et al., 2016), and there is increasing evidence for the existence of pathway-specific metabolons (Bassard and Halkier, 2017). Within the crowded intracellular environment where proteins are constantly coming into physical contact, it is an open question how multi-enzyme pathways are orchestrated in response to developmental and exogenous cues. Are they self-assembled, or are scaffolding proteins and chaperones involved? Additionally, how do specific pathways couple to surrounding protein-protein interaction networks within the cell?

The glucosinolate defense compounds are suitable as model metabolites for studying pathway orchestration, as they are present in the model plant *Arabidopsis thaliana* (hereafter Arabidopsis) that offers extensive 'omics databases, bioinformatics tools as well as large mutant collections. In Arabidopsis, the glucosinolates are present as methionine-derived aliphatic and tryptophan-derived indole glucosinolates. All of the enzymes in the biosynthetic pathways are known (Sønderby et al., 2010). The respective amino acid is converted to a glucosinolate in seven consecutive enzymatic reactions constituting the core pathway. The first two steps are carried out by different endoplasmic reticulum (ER) membrane-bound cytochrome P450s, CYP79s and CYP83s, followed by five steps catalyzed by the cytoplasmic enzymes GSTs, GGP1, SUR1, UGT74s and SOTs (Figure S1). Some of these enzymes, e.g., the soluble SUR1 and GGP1, are shared between the two pathways, whereas others such as the CYP79s and CYP83s are specific for the respective class of glucosinolates. In addition to the core pathway, formation of aliphatic glucosinolates requires chain-elongation of the precursor methionine, which is a process catalyzed by plastid-localized enzymes.

The glucosinolate pathways are proposed to be organized in metabolons (Halkier and Gershenzon, 2006). An unstable pathway intermediate, *S*-alkyl thiohydroximate, produced by GGP1, will intramolecularly cyclize and hence result in abortion of the pathway, if the intermediate is not further metabolized immediately. This suggests a tight coupling between at least GGP1 and the following SUR1 (Mikkelsen et al., 2004). Furthermore, as cytochrome P450 enzymes are typically ER membrane-anchored, it has been speculated that the P450s can serve as nucleation points that recruit the soluble enzymes to the ER surface by protein-protein interactions (Jørgensen et al., 2005; Ralston and Yu, 2006). In addition, UGT74B1 enzyme kinetics support a channeling mechanism to achieve efficient reaction speed at physiological substrate concentration and to avoid product inhibition (Kopycki et al., 2013). Indeed, investigations of specific protein-protein interactions within the pathway have revealed interactions between the UGT74 and SOT enzymes, independently confirmed by yeast 2-hybrid, bimolecular fluorescence complementation (BiFC) and Förster resonance energy transfer (FRET) measurements (Andersen et al., 2016).

Besides the intra-pathway interactions discussed above, several proteins that are not involved in glucosinolate biosynthesis interact with glucosinolate biosynthetic enzymes. CYP83A1—in aliphatic glucosinolate biosynthesis—has several reported interactors (Manzano et al., 2008; Lalonde et al., 2010; Weis et al., 2013; Jones et al., 2014). Its interaction with the BAX INHIBITOR 1 (BI1) protein, which has a function in programmed cell death and response to pathogen infection (Weis et al., 2013), has been further investigated, and it was speculated that this interaction facilitates the assembly or stabilization of a glucosinolate biosynthetic enzyme complex (Weis et al., 2014). CYP83B1—in tryptophan-derived glucosinolate biosynthesis—is reported to interact with a gibberellin receptor that binds to DELLA proteins (Griffiths et al., 2006; Middleton et al., 2012). The role of this interaction is unknown, but it might play a role in balancing growth and defense responses. Collectively, these findings suggest that the enzymes in the glucosinolate pathways are subject to diverse protein-protein interactions that crucially contribute to their regulation and interconnection with signaling pathways and cellular metabolism.

Toward our goal to understand pathway orchestration and to link the glucosinolate pathways to their underlying protein-protein interaction networks, we screened for protein interactors in two types of untargeted screens. As baits, we used the pathway-specific CYP83A1 and CYP83B1 that catalyze steps in aliphatic or indole glucosinolate biosynthesis, respectively. These are the only enzymes that can be non-redundantly assigned to either the aliphatic or indole core pathway (Bak and Feyereisen, 2001; Hansen et al., 2001; Naur et al., 2003; Sønderby et al., 2010). To maximize coverage, we performed several split-ubiquitin-based yeast 2-hybrid screens (Stagljar et al., 1998; Möckli et al., 2007) as well as complementary co-immunoprecipitation (Co-IP) assays followed by tandem mass spectrometry (MS/MS)-based protein identification. Despite little overlap in detected protein-protein interactions between the conducted screens, the detected proteins overlap functionally. Network analysis revealed that the two glucosinolate biosynthetic pathways show differences in the way they interconnect with their cellular environment. As an example, we investigate a small family of hypersensitive response (HR)-like lesion-inducing proteins that interact with glucosinolate biosynthetic enzymes and provide a possible link to innate immunity.

## Results

### Candidates identified by tissue-specific split-ubiquitin yeast 2-hybrid screens

To identify the protein-protein interaction networks for the aliphatic and indole glucosinolate pathways, we conducted split-ubiquitin-based yeast 2-hybrid screens using CYP83A1 and CYP83B1 tagged with the C-terminal part of ubiquitin as baits. We constructed cDNA prey libraries from tissues with particularly high *CYP83A1* and *CYP83B1* transcript levels, respectively, to enrich for cDNAs of naturally co-occurring proteins. Based on *CYP83* transcript levels available in the eFP browser of the BAR database (Winter et al., 2007), cDNA libraries were generated from node tissue (center of rosettes of 3-week-old plants, high in *CYP83A1* transcripts) and hypocotyl tissue (from 10-day-old plants, high in *CYP83B1* transcripts) of Arabidopsis ecotype Col-0. To investigate the importance of the tissue context, both libraries were screened with the CYP83A1 and CYP83B1 baits.

We analyzed plasmids from 16 (CYP83A1 with hypocotyl library), 144 (CYP83A1 with node library), 25 (CYP83B1 with hypocotyl library) and 56 (CYP83B1 with node library) yeast colonies by sequencing. Using the basic local alignment search tool (BLAST) of The Arabidopsis Information Resource (TAIR, www.arabidopsis.org), we identified a total of 72 unique inserts, most of which represent full length or nearly full length coding sequences. Duplicates and sequences unambiguously belonging to proteins of the photosynthetic and the ribosomal machinery were excluded from further analysis, leaving a curated list of 60 candidate interactors (Table 1).

**Table 1:** Curated list of candidate interactors found in untargeted yeast 2-hybrid library screens.

**Gene**	**Description**	**MW (kDa)**	**TM domains**	**Location**	**CYP83A1/Node**	**CYP83A1/Hypocotyl**	**CYP83B1/Node**	**CYP83B1/Hypocotyl**
At1G04750.1	Vesicle associated membrane protein 721 (VAMP721)	24.8	1	Cell membrane	1	–	–	–
At1G06090.1	Fatty acid desaturase family	34.8	5	ER membrane	–	–	1	–
At1G15500.1	ATP:ADP antiporter (NTT2)	67.5	11	Plastid membrane	–	–	1	–
At1G16890.2	Ubiquitin-conjugating enzyme 36 (UBC36)	17.2		na	1	–	–	–
At1G26670.1	Vesicle transport V-SNARE 12 (VTI12)	25.1	1	Golgi network membrane	1	–	–	–
At1G28250.1	Unknown	11.2	2	na	–	–	1	–
At1G28490.1	Syntaxin of plants 61 (SYP61)	27.7	1	Golgi network membrane	2	–	–	–
At1G30935.1	F-box protein family (FBX1)	46.5	1	Membrane	1	–	–	–
At1G44920.1	Unknown	27.7	4	na	1	–	1	–
At1G52260.1	Protein disulfide isomerase-like, thioredoxin superfamily (PDI3)	60.2		ER	–	–	1	–
At1G52870.2	Mpv17/PMP22 family; Peroxisomal membrane protein	41.3		*Chloroplast*	–	–	1	–
At1G57765.1	Unknown	11.9	1	*Secreted*	–	–	1	2
At1G59890.2	SIN3-LIKE 5 (SNL5)	133.0		Nucleus	–	–	1	–
At1G66240.1	Homolog of anti-oxidant 1 (ATX1)	8.2		Cytosol	–	–	1	–
At1G68220.1	Unknown	21.4	4	*Secreted*	1	–	-	–
At2G05630.1	ATG8D	13.9		Cytoplasmic vesicle, Lipid anchor	1	–	–	–
At2G25610.1	ATPase, F0/V0 complex, subunit C protein;	18.2	4	ER membrane	2	1	–	–
At2G28900.1	Outer envelope protein 16 (OEP16)	15.5	3	Plastid outer membrane	1	–	–	–
At2G32380.1	Transmembrane protein 97	18.3	4	*Secreted*	1	–	–	–
At2G32720.1	Cytochrome B5-B (CB5-B)	15.0	1	ER membrane	1	–	–	–
At2G33120.1	Vesicle associated membrane protein 722 (VAMP722)	24.9	1	Cell membrane	2	–	–	–
At2G41110.1	Calmodulin 2, AtCAL5 (CAM2)	16.8		Cytoplasm, cytoskeleton	1	–	–	–
At3G12120.1	Fatty acid desaturase 2 (FAD2)	44.0	5	ER membrane	1	1	–	–
At3G12870.1	Unknown	23.7	3	*Other (e.g., cytoplasm)*	–	–	1	–
At3G13520.1	Arabinogalactan protein 12 (AGP12)	6.1		Cell membrane (GPI-anchor)	–	–	1	–
At3G15820.1	Reduced oleate desaturation 1, phosphatidylcholine:diacylglycerol cholinephosphotransferase (ROD1)	33.0	5	ER membrane	1	–	–	–
At3G18280.1	Bifunctional inhibitor/lipid-transfer protein/seed storage 2S albumin superfamily protein	10.3		*Secreted*	–	–	–	1
At3G48990.1	Acyl-activating enzyme 3 (AAE3)	55.5		Cytoplasm	–	–	–	1
At3G50685.1	Unknown	16.1	3	na	–	–	1	–
At3G51460.1	Root hair defective 4 (RHD4)	68.2	2	ER membrane	–	1	–	–
At3G60600.1	VAMP/Synaprobrevin-associated protein 27-1, Vesicle associated protein (VAP27-1)	28.5	1	ER membrane	–	1	–	–
At4G01150.1	Curvature thylakoid 1A (CURT1A)	17.7	2	Plastid, chloroplast	1	–	–	–
At4G14420.1	HR-like lesion-inducing protein-related	17.4	2	*Secreted*	2	1	–	–
At4G16410.1	Unknown	20.4	3	na	4	–	1	–
At4G24920.1	Sec61 subcomplex, protein transmembrane transporter	7.7	1	ER membrane	1	–	2	–
At4G27500.1	Proton pump interactor 1 (PPI1)	68.9	1	Cell membrane	–	3	–	–
At4G30950.1	Fatty acid desaturase 6 (FAD6)	51.2	4	Plastid inner membrane	–	–	1	–
At4G31840.1	Early nodulin-like protein 15 (ENODL15)	19.0	1	*Extracellular*	4	3	7	3
At5G14330.1	Unknown	14.2	2	na	1	–	–	–
At5G14720.1	Protein kinase superfamily	75.4		*Other (e.g., cytoplasm)*	–	–	1	–
At5G16830.1	Syntaxin of plants 21 (SYP21)	31.1	1	Prevacuolar compartment	–	–	–	2
At5G20130.1	Unknown	21.9		na	1	–	–	–
At5G24680.1	Peptidase C78, ubiquitin fold modifier-specific peptidase 1/ 2	44.3		na	4	–	–	–
At5G38660.1	Acclimation of photosynthesis to environment (APE1)	31.4	1	*Plastid*	–	–	1	–
At5G39510.1	Vesicle transport V-SNARE 11 (VTI11)	25.0	1	Golgi network membrane	1	–	–	–
At5G42000.1	ORMDL family protein	18.1	3	*Other (e.g., cytoplasm)*	2	–	–	–
At5G42570.1	B-cell receptor-associated 31-like	24.6	3	*Secreted*	–	–	1	–
At5G43460.1	HR-like lesion-inducing protein-related	16.9	3	*Secreted*	63	6	17	6
At5G43580.1	Unusual serine protease inhibitor (UPI)	11.1		na	–	–	–	1
At5G43970.1	Translocase of outer membrane 22-V, TOM9-2 (TOM22-V)	10.4	1	Mitochondrion outer membrane	–	–	5	-
At5G45420.1	Membrane anchored MYB (maMYB)	34.4	2	*Other (e.g., cytoplasm)*	1	1	2	1
At5G45680.1	FK506 binding protein 13, peptidyl-prolyl isomerase (FKBP13)	22.0		Plastid	–	1	–	–
At5G50740.3	Heavy metal transport/detoxification superfamily protein	32.9		*Other (e.g. cytoplasm)*	–	–	–	1
At5G51010.1	Rubredoxin-like superfamily	17.2	1	na	2	–	1	–
At5G51400.1	PLAC8 family	27.0		*Other (e.g., cytoplasm)*	–	–	–	1
At5G52240.1	Membrane-associated progesterone binding protein 5, ATMP1, Membrane steroid binding protein 1 (MSBP1)	24.4	1	Cell membrane	–	1	–	–
At5G52980.1	ATPase, vacuolar ER assembly factor, Vma12	24.5	2	na	–	–	1	–
At5G60920.1	COBRA (COB)	51.2		Cell membrane, Lipid anchor	1	–	–	–
At5G61790.1	Calnexin 1 (CNX1)	60.5	1	ER membrane	1	–	–	–
At5G67600.1	Windhose 1 (WIH1)	8.7		*Other (e.g., cytoplasm)*	4	–	–	–

*Gene models and descriptions are based on The Arabidopsis Information Resource (TAIR10, www.arabidopsis.org). Information about the molecular weight (MW) and transmembrane (TM) domains were obtained from the UniProt databases (Bateman et al., 2015; Boutet et al., 2016). Information and predictions of protein locations are based on UniProt databases (regular font) or the SUBA3 database (italic font) (Hooper et al., 2014). The columns to the right indicate occurrences of sequenced plasmids containing the respective sequences in the four screens with combinations of CYP83A1 or CYP83B1 as baits and libraries constructed from node and hypocotyl tissue. na, not available*.

Most sequences were found only once, with few sequences identified several times, and one coding sequence found in 93 out of the total 241 sequenced plasmids (Figure S2A). PANTHER Gene Ontology (GO)-Slim biological processes *protein localization* and *organelle organization* were over-represented in the detected interactors of CYP83A1, while no over-representation was found for the interactors of CYP83B1.

Of the 60 identified proteins, 30 were exclusively interacting with CYP83A1, while 23 proteins were only identified in the CYP83B1 screens (Figure S2B). Seven were found in screens with both CYP83A1 and CYP83B1, including the candidate with the highest number of total occurrences in our screens, At5G43460, annotated as HR-like lesion-inducing protein-related. The choice of cDNA library had an impact on the total number of identified candidates in accordance with the determined library transformation titer (Table S2), with overall more sequences found employing the library constructed from node tissue and with a low overlap of interactors found in both libraries with each bait.

Because of the small overlap between interactors found with a single bait with different libraries and the relatively high number of candidates found with both baits, we investigated the specificity of the interactions for 13 of the proteins in targeted yeast 2-hybrid, three of which were identified using both CYP83 baits, while ten had been found exclusively with either CYP83A1 or CYP83B1 as bait. Their coding sequences were inserted into the prey vector and interaction with CYP83A1, CYP83B1 and the unrelated control bait protein LargeT was assessed (Figure S3). We found that all the tested preys interacted with both CYP83A1 and CYP83B1, and that seven of them also interacted with LargeT.

### Candidate interacting proteins identified by Co-IP

To identify a broad array of relevant interactors, we additionally performed Co-IP experiments followed by MS/MS-based protein identification as a complementary, principally different approach to our yeast 2-hybrid screens. Transgenic Arabidopsis lines expressing either CYP83A1 or CYP83B1 fused to the mVenus fluorescent protein driven by the respective native promoter enabled us to use the protein tag as epitope for Co-IP. Subjecting protein extracts from 14-day-old seedlings to our analysis identified a total of 1840 proteins. As selection criteria, potential interactors of CYP83A1 and CYP83B1 had to be identified with at least 5 spectra and to have 5-fold more spectral counts in the respective affinity enrichments compared to the wildtype control. We identified 38 and 40 candidates for CYP83A1 and CYP83B1, respectively, while four candidates were found with both bait proteins (Tables 2, 3). In our experiment, the CYP83A1-mVenus and CYP83B1-mVenus Co-IPs serve as control for each other, and the low overlap between detection of interactors excludes a frequent occurrence of proteins interacting with the fluorescent protein tag instead of the cytochrome P450 bait.

**Table 2:** Curated list of candidate interactors found by Co-IP with CYP83A1-mVenus.

**Gene**	**Description**	**MW (kDa)**	**TM domains**	**Location**	**Exp1**	**Exp2**	**Exp3**	**Total specs**
At1G11750	CLP protease proteolytic subunit 6 (CLPP6)	29.4		Plastid, stroma	2	0	5	7
At1G14810	Semialdehyde dehydrogenase family protein	40.7		*Plastid*	2	0	4	6
At1G20580	Small nuclear ribonucleoprotein family protein (SMD3)	14.2		Cytosol	7	4	0	11
At1G54100	Aldehyde dehydrogenase 7B4 (ALDH7B4)	54.2		*Cytosol*	1	0	5	6
At1G68830	STT7 homolog (STN7)	63.3		Plastid, thylakoid membrane	0	0	5	5
At1G70730	Phosphoglucomutase/phosphomannomutase family protein (PGM2)	63.5		Cytoplasm	3	0	4	7
At2G18960	H(+)-ATPase 1 (HA1)	104.2	10	Cell membrane	3	0	3	6
At2G40490	Uroporphyrinogen decarboxylase (HEME2)	43.6		Plastid	2	0	3	5
At3G09200	Ribosomal protein L10 family protein	34.1		*Cytosol*	6	0	6	12
At3G19170	Presequence protease 1 (PREP1)	121.0		Plastid, stroma, Mito., matrix	6	1	7	14
At3G22142	Encodes a Protease inhibitor/seed storage/LTP family protein	147.2		*Secreted*	8	0	0	8
At3G26070	Plastid-lipid associated protein PAP / fibrillin family protein;	27.2		Plastid	0	0	6	6
At3G48000	Aldehyde dehydrogenase 2 (ALDH2)	58.6		Mitochondrion, matrix	5	0	10	15
At3G48420	Haloacid dehalogenase-like hydrolase superfamily protein	34.2		Plastid	5	0	1	6
At3G56240	Copper chaperone (CCH)	13.0		*Cytosol*	4	2	2	8
At3G57610	Adenylosuccinate synthase (ADSS)	53.0		Plastid	3	0	3	6
At3G58730	Vacuolar ATP synthase subunit D (VATPD)	29.1		Vacuole membrane	0	0	5	5
At3G61440	Cysteine synthase C1 (CYSC1)	39.9		Mitochondrion	5	2	8	15
At4G13770	Cytochrome P450, family 83, subfamily A, polypeptide 1 (CYP83A1)	57.4	1	ER membrane	157	37	34	228
At4G15530	Pyruvate orthophosphate dikinase (PPDK)	105.1		Cytosol, Plastid	0	0	7	7
At4G22240	Plastid-lipid associated protein PAP / fibrillin family protein	33.7		Plastid	1	0	5	6
At4G23100	Glutamate-cysteine ligase (GSH1)	58.6		Plastid	3	0	3	6
At4G23600	Tyrosine transaminase family protein (CORI3)	47.0		*Cytosol*	0	2	11	13
At4G25080	Magnesium-protoporphyrin IX methyltransferase (CHLM)	33.8		Plastid, peripheral membrane	1	0	5	6
At4G30270	Xyloglucan endotransglucosylase/hydrolase 24 (XTH24)	30.8		Secreted	0	0	5	5
At4G33680	Pyridoxal phosphate-dependent transferases superfam. prot. (AGD2)	50.4		Plastid	3	0	2	5
At5G11520	Aspartate aminotransferase 3 (ASP3)	49.0		Plastid	1	0	4	5
At5G11670	NADP-malic enzyme 2 (NADP-ME2)	64.4		Cytoplasm	5	0	1	6
At5G14200	Isopropylmalate dehydrogenase 1 (IMD1)	44.2		Plastid	3	0	5	8
At5G14780	Formate dehydrogenase (FDH)	42.4		Mitochondrion	6	1	7	14
At5G17710	Co-chaperone GrpE fam. protein/embryo defective 1241 (EMB1241)	35.5		Mitochondrion, matrix	6	1	4	11
At5G19550	Aspartate aminotransferase 2 (ASP2)	44.3		Cytoplasm	1	0	5	6
At5G38660	Acclimation of photosynthesis to environment (APE1)	31.4	1	*Plastid*	8	0	1	9
At5G42650	Allene oxide synthase (AOS)	58.2		Plastid	6	0	15	21
At5G44130	FASCICLIN-like arabinogalactan protein 13 precursor (FLA13)	26.2		Cell membrane, Lipid anchor	2	0	3	5
At5G49360	Beta-xylosidase 1 (BXL1)	83.5		Secreted	0	0	11	11
At5G51820	Phosphoglucomutase (PGM1)	68.0		Plastid	6	2	9	17
At5G60600	4-hydroxy-3-methylbut-2-enyl diphosphate synthase (HDS)	82.3		Plastid, stroma	1	0	10	11

*Gene identifiers and descriptions are based on The Arabidopsis Information Resource (TAIR10, www.arabidopsis.org). Information about the molecular weight (MW) and transmembrane (TM) domains were obtained from the UniProt databases (Bateman et al., 2015; Boutet et al., 2016). Information and predictions of protein locations are based on UniProt databases (regular font) or the SUBA3 database (italic font) (Hooper et al., 2014). Columns Exp and total specs show the number of recorded spectra in each of the three experiments or the sum thereof, respectively*.

**Table 3:** Curated list of candidate interactors found by Co-IP with CYP83B1-mVenus.

**Gene**	**Description**	**MW (kDa)**	**TM domains**	**Location**	**Exp1**	**Exp2**	**Exp3**	**Total specs**
At1G15280	CASC3/Barentsz eIF4AIII binding	63.3		*Nucleus*	3	3	0	6
At1G17220	Translation initiation factor 2, small GTP-binding protein (FUG1)	109.7		Plastid	2	7	0	9
At1G19120	Small nuclear ribonucleoprotein family protein	14.7		Cytosol	7	0	0	7
At1G19880	Regulator of chromosome condensation family protein	57.8		*Nucleus*	2	15	6	23
At1G20580	Small nuclear ribonucleoprotein family protein	14.2		Cytosol	12	5	2	19
At1G31280	Argonaute family protein (AGO2)	113.4		*Nucleus*	15	7	0	22
At1G50520	Cytochrome P450, family 705, subf. A, polypeptide 27 (CYP705A27)	60.6	1	*ER*	5	1	5	11
At1G66260	RNA-binding family protein	31.3		Nucleus, nucleoplasm	8	2	0	10
At1G67170	Unknown	39.7		*Nucleus*	11	0	0	11
At1G76300	snRNP core protein	13.8		Cytosol	3	3	1	7
At1G79090	Homolog of yeast PAT1	88.8		*Nucleus*	24	0	0	24
At1G79280	Nuclear pore anchor (NUA)	237.0		Nuclear envelope	0	24	0	24
At2G04170	TRAF-like family protein	44.4		*Cytosol*	11	0	0	11
At2G18960	H(+)-ATPase 1 (HA1)	104.2	10	Cell membrane	3	0	2	5
At2G20950	Arabidopsis phospholipase-like protein (PEARLI 4) family	59.6		*Nucleus*	5	0	0	5
At2G26280	CTC-interacting domain 7 (CID7)	62.1		*Pastid*	11	0	0	11
At2G30260	U2 small nuclear ribonucleoprotein B (U2B0)	26.2		Nucleus	3	4	0	7
At2G34040	Apoptosis inhibitory protein 5 (API5)	61.6		*Cytosol*	1	6	0	7
At2G37550	ARF-GAP domain 7 (AGD7)	49.3		Golgi apparatus	3	1	4	8
At2G44790	Uclacyanin 2 (UCC2)	20.4		Cell membrane, Lipid anchor	0	1	4	5
At3G04290	Li-tolerant lipase 1 (LTL1)	40.1		Secreted	5	0	0	5
At3G09200	Ribosomal protein L10 family protein	34.1		*Cytosol*	6	0	1	7
At3G11500	Small nuclear ribonucleoprotein family protein	8.8		*Cytosol*	4	1	1	6
At3G14750	Unknown	36.7		*Plastid*	8	0	0	8
At3G20550	Nuclear localized FHA (forhkead) domain containing protein	37.0		Nucleus	1	4	0	5
At3G22142	Protease inhibitor/seed storage/LTP family protein	147.2		*Extracellular*	7	2	0	9
At3G22270	Topoisomerase II-associated protein (PAT1H1)	85.7		*Nucleus*	15	1	0	16
At3G22430	Unknown	57.1		*Nucleus*	6	0	0	6
At3G54230	Suppressor of abi3-5 (SUA)	112.6		Nucleus	11	0	0	11
At3G62200	Putative endonuclease or glycosyl hydrolase	74.2		*Nucleus*	13	1	0	14
At4G12080	AT-hook motif nuclear-localized protein 1 (AHL1)	37.3		Nucleus, nucleoplasm	5	0	0	5
At4G14990	Topoisomerase II-associated protein (PAT1H2)	86.5		*Nucleus*	14	5	1	20
At4G16830	Hyaluronan/mRNA binding family	37.5		Cytoplasm, perinuclear	5	16	0	21
At4G20440	Small nuclear ribonucleoprotein associated protein B (smB)	27.1		Nucleus	4	1	0	5
At4G31500	Cytochrome P450, family 83, subfamily B, polypeptide 1 (CYP83B1)	56.8	1	Membrane	215	86	84	385
At5G04280	RNA-binding fam. protein w. retrovirus zinc finger-like dom. (RZ-1c)	33.5		Nucleus	24	0	1	25
At5G19950	Domain of unknown function	48.5		*Nucleus*	9	2	0	11
At5G23010	Methylthioalkylmalate synthase 1 (MAM1)	55.1		Plastid	0	0	5	5
At5G44070	Phytochelatin synthase 1, cadmium sensitive 1 (PCS1)	54.5		*Cytosol, nucleus*	9	1	0	10
At5G47430	DWNN domain, a CCHC-type zinc finger	99.1		Nucleus	4	1	0	5

*Gene identifiers and descriptions are based on The Arabidopsis Information Resource (TAIR10, www.arabidopsis.org). Information about the molecular weight (MW) and transmembrane (TM) domains were obtained from the UniProt databases (Bateman et al., 2015; Boutet et al., 2016). Information and predictions of protein locations are based on UniProt databases (regular font) or the SUBA3 database (italic font) (Hooper et al., 2014). Columns Exp and total specs show the number of recorded spectra in each of the three experiments or the sum thereof, respectively*.

Among the proteins detected to interact with CYP83A1 was Glutamate-cysteine ligase (GSH1 or PAD2, At4G23100), an enzyme in glutathione (GSH) biosynthesis (Parisy et al., 2007). Glutathione serves as sulfur donor for glucosinolate biosynthesis and accordingly, a *pad2* mutant had 80% reduced GSH levels and was affected in the synthesis of both aliphatic and indole glucosinolates (Schlaeppi et al., 2008). Functional categorization using all proteins quantified in the Co-IP experiment with the results of our CYP83A1 Co-IP as reference list and without correction for multiple testing revealed an enrichment of *porphyrin-containing compound* and *polysaccharide metabolic processes*. The enriched GO biological processes resulting from an analogous analysis for CYP83B1 interactors included *RNA metabolic and catabolic processes*.

### Yeast 2-hybrid and Co-IP identified complementary types of proteins

We observed almost no overlap between the candidate lists generated by Co-IP and by the yeast 2-hybrid screens. To complement our results, we integrated previously reported interactions from the BioGrid database (Chatr-Aryamontri et al., 2015) into our lists. Only two candidate proteins were present in more than one of these datasets, namely the membrane steroid-binding protein 1 (MSBP1, At5G52240), which was identified with the CYP83A1 bait in our yeast 2-hybrid screen and had previously been reported as an interactor of CYP83A1 via a protein-fragment complementation assay (Jones et al., 2014), and Acclimation of Photosynthesis to Environment (APE1, At5G38660), found in our screens as an interactor of CYP83B1 in yeast 2-hybrid and as an interactor of CYP83A1 in Co-IP (Figure S4).

**Figure 1:**
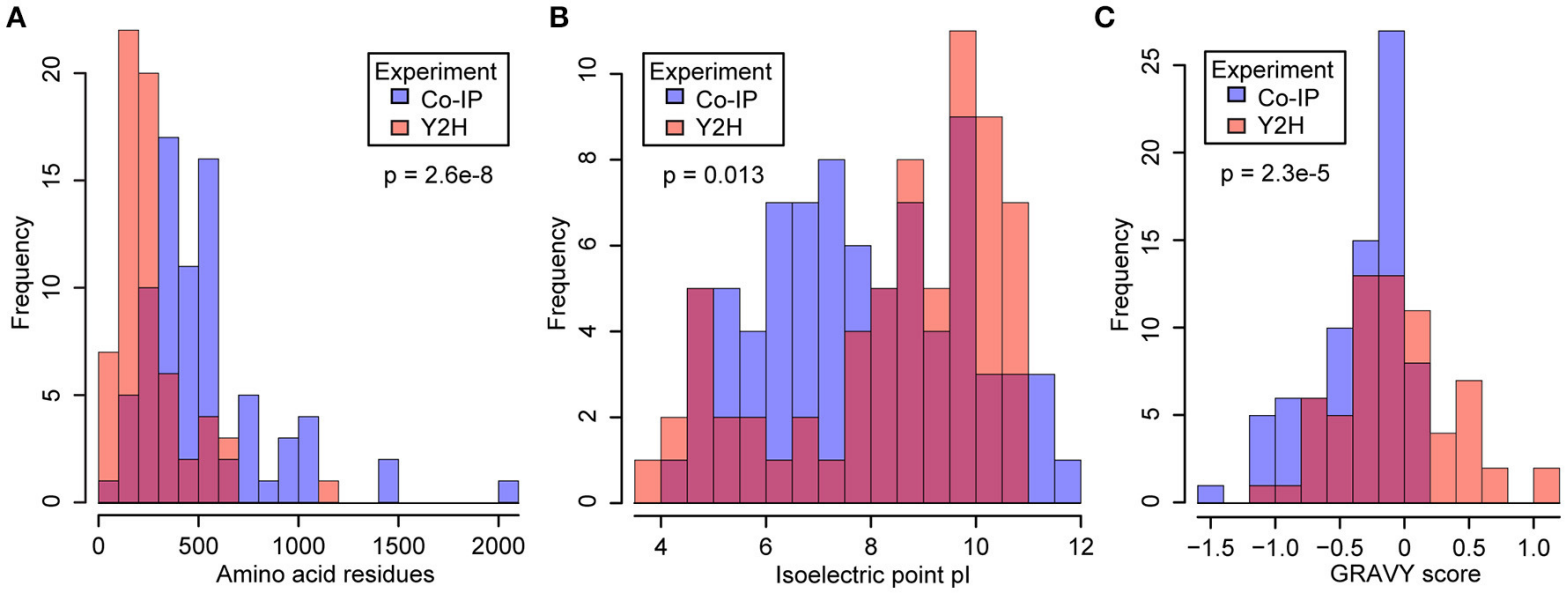
Comparison of physical and chemical properties of proteins identified by yeast 2-hybrid (Y2H) and co-immunoprecipitation (Co-IP). Y2H, orange bars; Co-IP, blue bars; overlap, red. Protein lengths **(A)**, isoelectric points **(B)** and hydrophobicity values as GRAVY scores **(C)** were obtained from the SUBA3 database (Hooper et al., 2014). The significance of the difference was tested by Welch two-sample *t*-tests **(A,C)** and a two-sample Kolmogorov-Smirnov test **(B)**.

Due to the complementary nature of the two methods, they are not expected to identify a large number of common candidates. To address this, we compared the physical-chemical properties of the identified proteins. The yeast 2-hybrid approach identified comparatively short proteins with an average length of 257.7 amino acids, and thereby shorter than the average length of 402.4 amino acids for all Arabidopsis proteins in TAIR10. With an average length of 486.7 amino acids for CYP83A1 interactors and of 556.83 for CYP81B1 interactors, the Co-IP-identified proteins had overall longer primary sequences (Figure 1A). Additionally, we compared the isoelectric points of the candidates identified by the two methods. Co-IP-identified proteins showed pI values between 12 and 4, with an average of 7.4 for CYP83A1 interactors and 8.1 for CYP83B1 interactors. The candidates identified by yeast 2-hybrid showed a tendency toward either higher or lower isoelectric points, with an average pI of 8.2 and 63% of them having a pI between 8 and 12 (Figure 1B). Under the cytosolic pH conditions of Arabidopsis (Shen et al., 2013), most yeast 2-hybrid candidates would thus be positively or negatively charged.

To compare the hydrophobicity of the candidate interactors detected with the two methods, we obtained the average hydropathy values (GRAVY scores) of the proteins as a measure for hydrophobicity (Kyte and Doolittle, 1982). It is evident that the proteins identified in our yeast 2-hybrid screens showed a balanced distribution of positive and negative scores. In contrast, the pulldown candidates showed predominantly negative GRAVY scores, indicative of a bias toward overall hydrophilic proteins (Figure 1C). The number of proteins containing transmembrane domains in each list (Tables 1–3) supports this, as less than 10% of the candidates from the Co-IP experiments contain predicted transmembrane domains, while as much as around 70% of the candidates identified via yeast 2-hybrid contain at least one predicted transmembrane domain, underlining the clear difference in hydrophobicity.

To assess a possible bias against lowly expressed proteins, especially in the identified Co-IP candidates, we extracted the average expression values of the respective transcripts in vegetative rosettes from the eFP anatomy browser of the BAR database (Winter et al., 2007). As we had observed previously that absolute protein and transcript levels correlate positively, transcript expression values can be taken as a proxy for protein abundance levels (Baerenfaller et al., 2008). The average log2-transformed transcript abundance was 8.94 for the CYP81A1 interactors and 6.98 for the CYP81B1 interactors, respectively, while it was 7.4 for the interactors identified with yeast 2-hybrid. Furthermore, the interactors identified with Co-IP did not show a correlation or a linear relationship between the number of identified spectra and transcript abundance (Figure S5). The Co-IP therefore also identified candidates with very low transcript levels and, correspondingly, expected low protein levels. Thus, the bias for rather highly expressed proteins usually observed in high-throughput proteomics experiments was not detected here.

### Identified candidates belong to different functional networks

To gain further insights into our datasets and to discover functional connections within the candidate interactor lists, we performed network analyses by submitting the candidate lists—together with the previously reported interactors from the BioGrid database—to network analysis using the STRING database (Szklarczyk et al., 2015). This analytical tool builds networks based on several reported factors, including physical interactions, but also other functional associations such as co-expression and participation in biological processes. The meta-analysis results in a score representing the connection between two proteins. In our analysis, we used a threshold score of 0.4.

**Figure 2:**
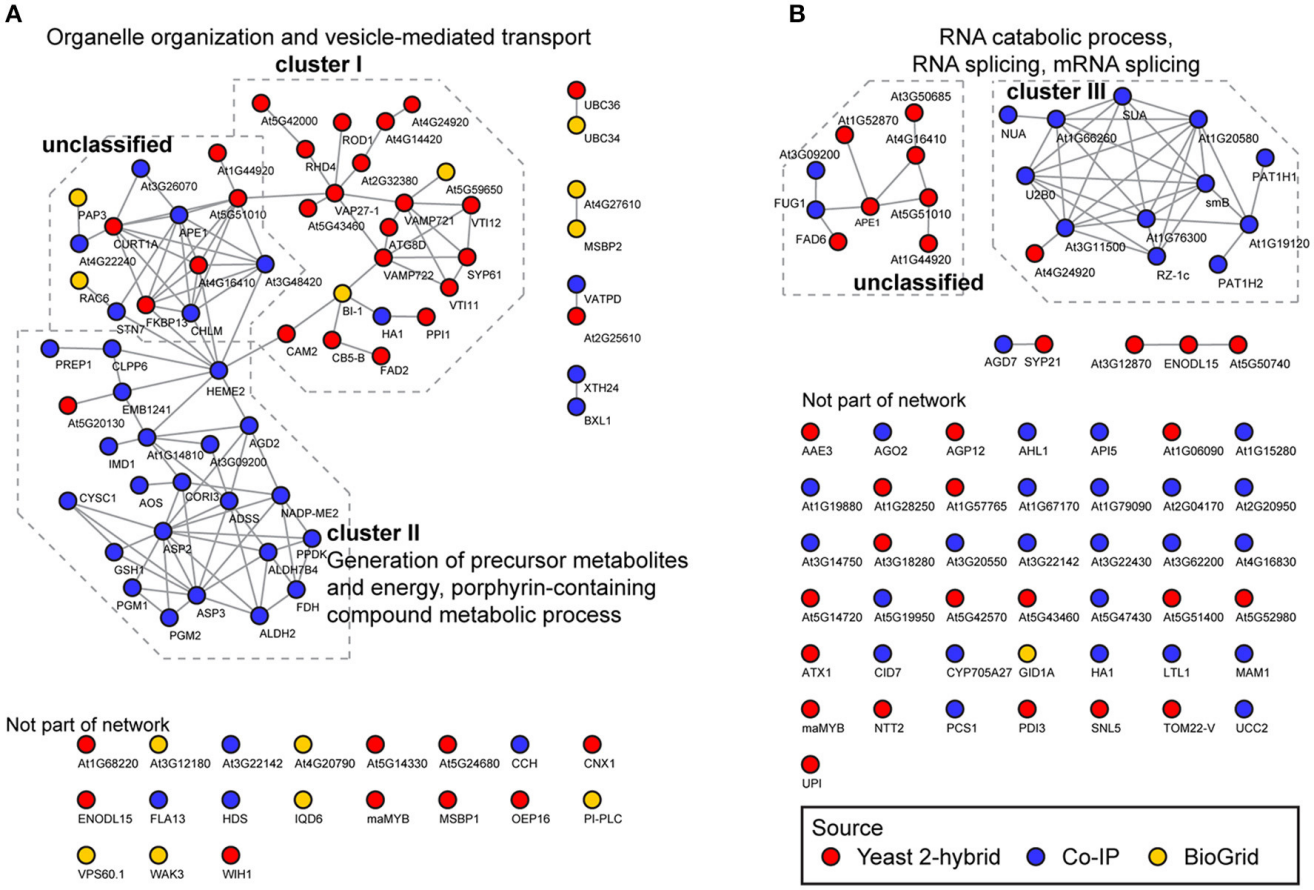
Functional networks of CYP83A1 and CYP83B1 interactors. Network analysis was performed on interactors identified by yeast 2-hybrid, Co-IP and the BioGrid database using CYP83A1 **(A)** and CYP83B1 **(B)** as baits. The network was built using the STRING database with a threshold score of 0.4 (Szklarczyk et al., 2015). Emerging clusters were subjected to Gene Ontology enrichment, and the main enriched GO categories of the respective sub-clusters are displayed.

For interactors of CYP83A1, this resulted in a functional network with 81 nodes and 115 connections, significantly above the expected values for a random sample (48 connections expected, interaction enrichment *p*-value = 2.22e-16; Figure 2A). Most proteins are included in two major subclusters. Cluster I consists mostly of candidate interactors detected in yeast 2-hybrid screens and is significantly enriched for GO categories *organelle organization* and *vesicle-mediated transport*. Interestingly, several interactors of the BI1 protein, which was previously reported to interact with CYP83A1, are present in cluster I, placing BI1 centrally in this cluster. Furthermore, the interaction of BI1 with CAM2 and HEME2 forms a direct link between clusters I and II. Cluster II contains proteins that were, apart from one exception, exclusively found in Co-IP, and GO categories *generation of precursor metabolites and energy*, as well as *porphyrin-containing compound metabolic process* are over-represented in this cluster.

The detected interactors of CYP83B1 form a network with 70 nodes and 45 connections (17 connections expected, interaction enrichment *p*-value = 2.04e-8; Figure 2B). Here, interactors mostly detected by Co-IP constitute a cluster with proteins enriched in GO categories *RNA catabolic process, RNA splicing, mRNA splicing, cellular component biogenesis* and *nitrogen compound metabolic process* (cluster III). A second, independent cluster consisting of yeast 2-hybrid as well as two Co-IP candidates is not significantly enriched for functional categories. In contrast to the CYP83A1 network, the majority of CYP83B1 interactors could not be functionally linked to either of the clusters. While our network analysis underscores the individual strengths of the employed methods in detecting proteins of largely differing characteristics, functional connections could be drawn within but also between the datasets obtained by the complementary methods.

### Glucosinolate profiles of mutant alleles of candidate interactors suggest biological functions impacting glucosinolate metabolism

**Figure 3:**
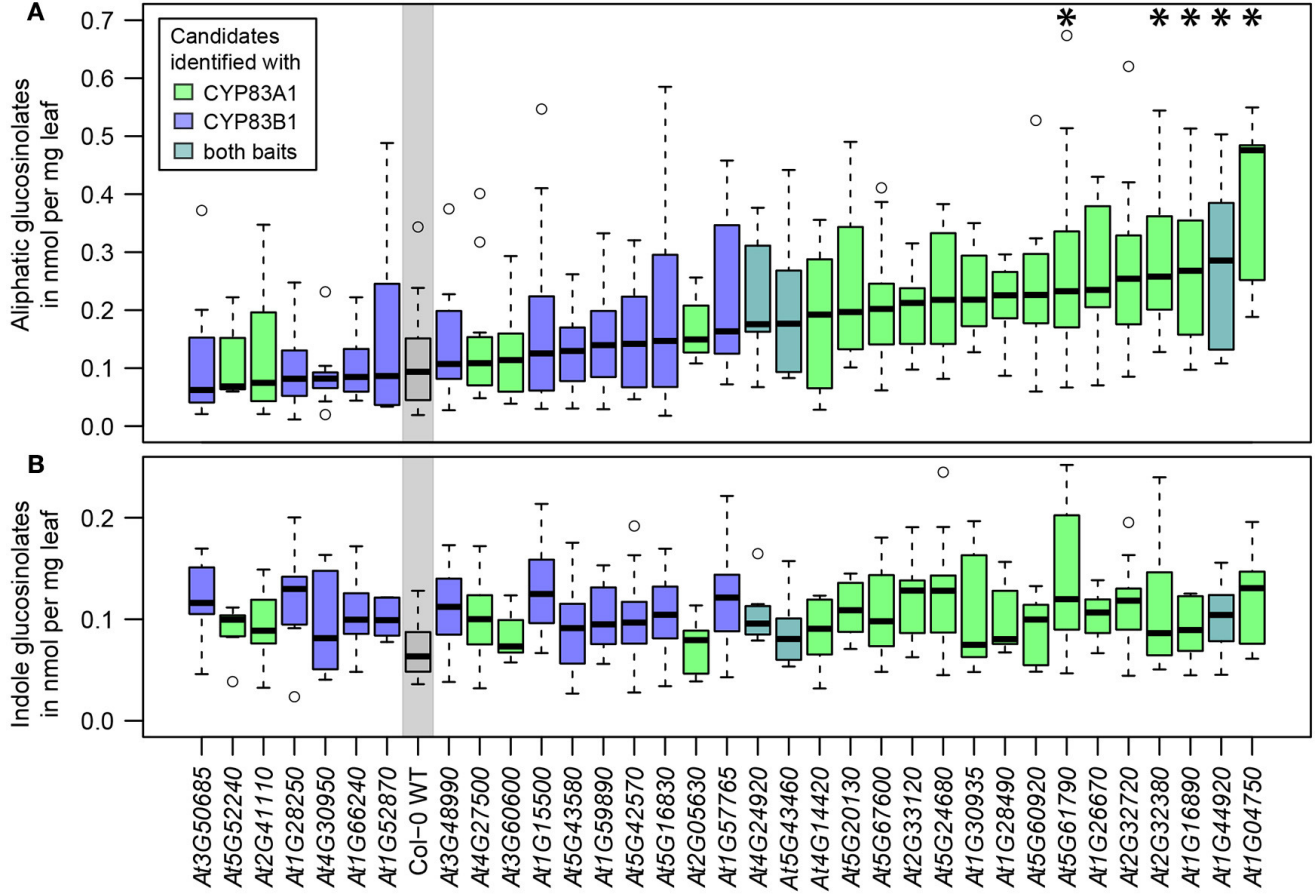
Glucosinolate analysis of plants carrying mutant alleles of identified genes. **(A)** Total aliphatic glucosinolate levels. **(B)** Total indole glucosinolate levels. The genes of candidate interactors are sorted and plotted according to their median aliphatic glucosinolate content and the plot is color coded according to the baits with which the proteins were identified. Glucosinolate levels significantly deviating from wildtype (WT) levels are marked with ^*^ [Two-way ANOVA (Table S4) followed by *post-hoc t*-test, Holm-adjustment for multiple testing, *p* < 0.05, *n* = 6–12, Col-0 WT: *n* = 22].

To assess the impact of the detected interactors on glucosinolate metabolism and thus assign an *in planta* function, we analyzed glucosinolate profiles of available homozygous SALK T-DNA insertion lines, targeting 34 of the 54 potential interactors identified with yeast 2-hybrid along with wildtype Arabidopsis (Figure 3, Table S3). No significant changes in indole glucosinolates were detected, while most tested T-DNA lines showed at least a tendency toward higher aliphatic glucosinolate levels, and five of these showed a significant increase in total aliphatic glucosinolate abundance. The respective lines contained insertions in genes coding for a vesicle associated membrane protein (VAMP721, At1G04750), a ubiquitin-conjugating enzyme (UBC36, At1G16890), Calnexin 1 (CNX1, At5G61790) and two unknown membrane proteins (At2G32380, At1G44920). Strikingly, all five lines with significantly increased aliphatic glucosinolates contained mutations in genes found with the CYP83A1 bait (Figure 3).

### Proteins associated with innate immunity interact with glucosinolate biosynthetic enzymes

**Figure 4:**
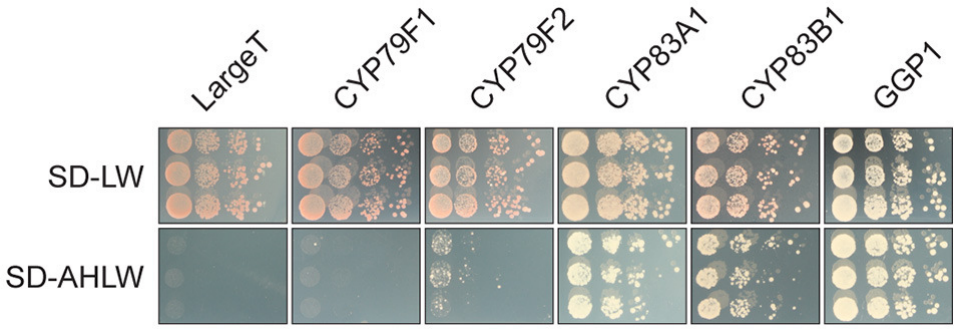
The At5G43460 protein interacts with several glucosinolate biosynthetic enzymes. Combinations of At5G43460 and enzymes from the glucosinolate biosynthetic pathways or the unrelated control bait protein LargeT were analyzed in a yeast 2-hybrid assay. Growth control (SD-LW medium) and interaction assay (SD-AHLW medium) is depicted.

Two homologous proteins annotated as HR-like lesion-inducing protein-related (At4G14420, At5G43460) were detected as interactors of CYP83A1 and, in the case of At5G43460, also of CYP83B1 in our yeast 2-hybrid screens. Strikingly, At5G43460 was found multiple times in all four of these screens, thus prompting us to investigate its interaction specificity and potential role in glucosinolate metabolism. In targeted assays, we re-tested the interaction between this protein and both CYP83 enzymes and investigated interactions with other glucosinolate biosynthetic enzymes, as well as the unrelated LargeT protein. We found that co-expressing At5G43460 with CYP83s, CYP79F2 or GGP1, but not CYP79F1 or LargeT enabled yeast cells to grow on selective medium (Figure 4). These results show that while the At5G43460 gene product interacts with several glucosinolate biosynthetic enzymes, it only activates the yeast 2-hybrid system when co-expressed with certain baits. We were thus interested in whether this protein or its homologs affect glucosinolate accumulation.

The gene products of At5G43460 and At4G14420 are annotated as HR-like lesion-inducing proteins-related, as they contain an HR-like lesion-inducer domain (PFAM:PF05514/InterPro:IPR008637). However, no molecular function has yet been assigned to these proteins. The Arabidopsis genome encodes four additional homologous proteins that share the HR-like lesion-inducer domain, one of them, At1G04340, is a close homolog of At5G43460 and At4G14420. We named the gene products of *At1G04340, At4G14420*, and *At5G43460* HR-like 1, 4, and 5, respectively, according to their gene locations on the different chromosomes. At the amino acid level, HR-like 5 shares 83 and 52% sequence identity with HR-like 1 and HR-like 4, respectively. The three proteins form a subclade within the small family of HR-like proteins (Figure S6). The other three family members, *At3G23175, At3G23180*, and *At3G23190* are located in close proximity on chromosome 3 and share between 19 and 24% amino acid identity with the members of the subclade with HR-like 1, 4, and 5. The high similarity of HR-like 1, 4, and 5 and their potential involvement in innate immunity prompted us to further investigate these proteins, despite the fact that mutations in individual genes did not appear to alter glucosinolate accumulation in the respective T-DNA insertion lines (Figure 3).

**Figure 5:**
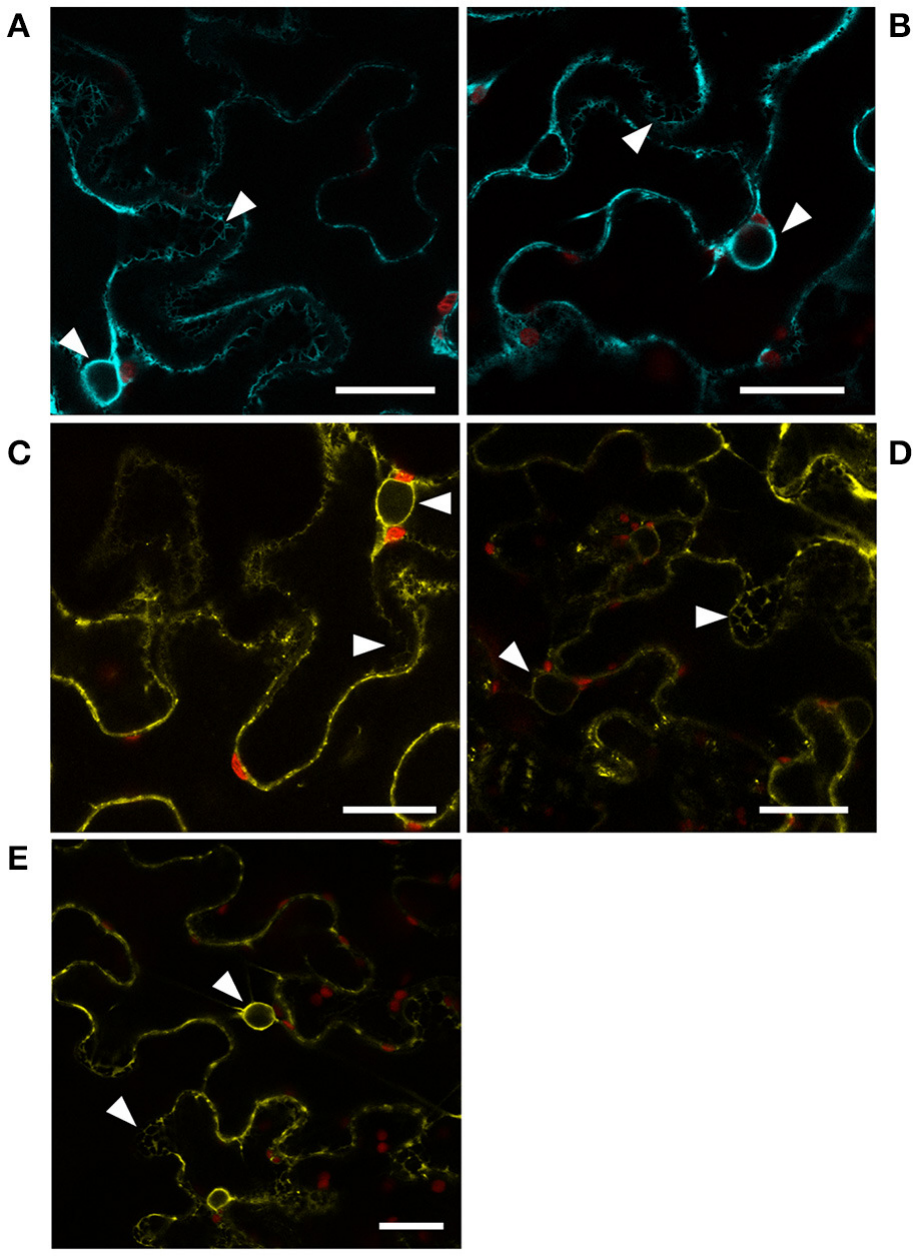
Localization of CYP83 enzymes and HR-like proteins. Subcellular localization of **(A)** CYP83A1-mTurquoise2, **(B)** CYP83B1-mTurquoise2, **(C)** HR-like 1-mVenus, **(D)** HR-like 4-mVenus, and **(E)** HR-like 5-mVenus fusion proteins transiently expressed in *Nicotiana benthamiana* epidermis cells and observed by confocal laser scanning microscopy. mTurquoise2 and mVenus fluorescence signal is represented by cyan and yellow, respectively, while red represents signal containing chlorophyll autofluorescence. White arrow heads point to signal from ER membranes in mesh-like structures and surrounding the nuclei. Scale bars: 25 μm.

To address whether protein-protein interactions between CYP83 enzymes and HR-like proteins could fulfill a biological function *in planta*, we transiently expressed fluorophore-tagged versions of CYP83A1 and CYP83B1 as well as HR-like 1, 4, and 5 in *Nicotiana benthamiana* epidermis cells to determine their subcellular localization (Figure 5). The visible mesh structure and the signal surrounding, but not filling the nuclei, confirmed the predicted presence of CYP83 enzymes at the ER membrane (Figures 5A,B). We observed the same pattern for all three investigated HR-like proteins (Figures 5C–E), indicating the presence of all five proteins in the ER membrane, a prerequisite for protein-protein interactions to occur *in planta*.

### HR-like proteins have an impact on glucosinolate accumulation

**Figure 6:**
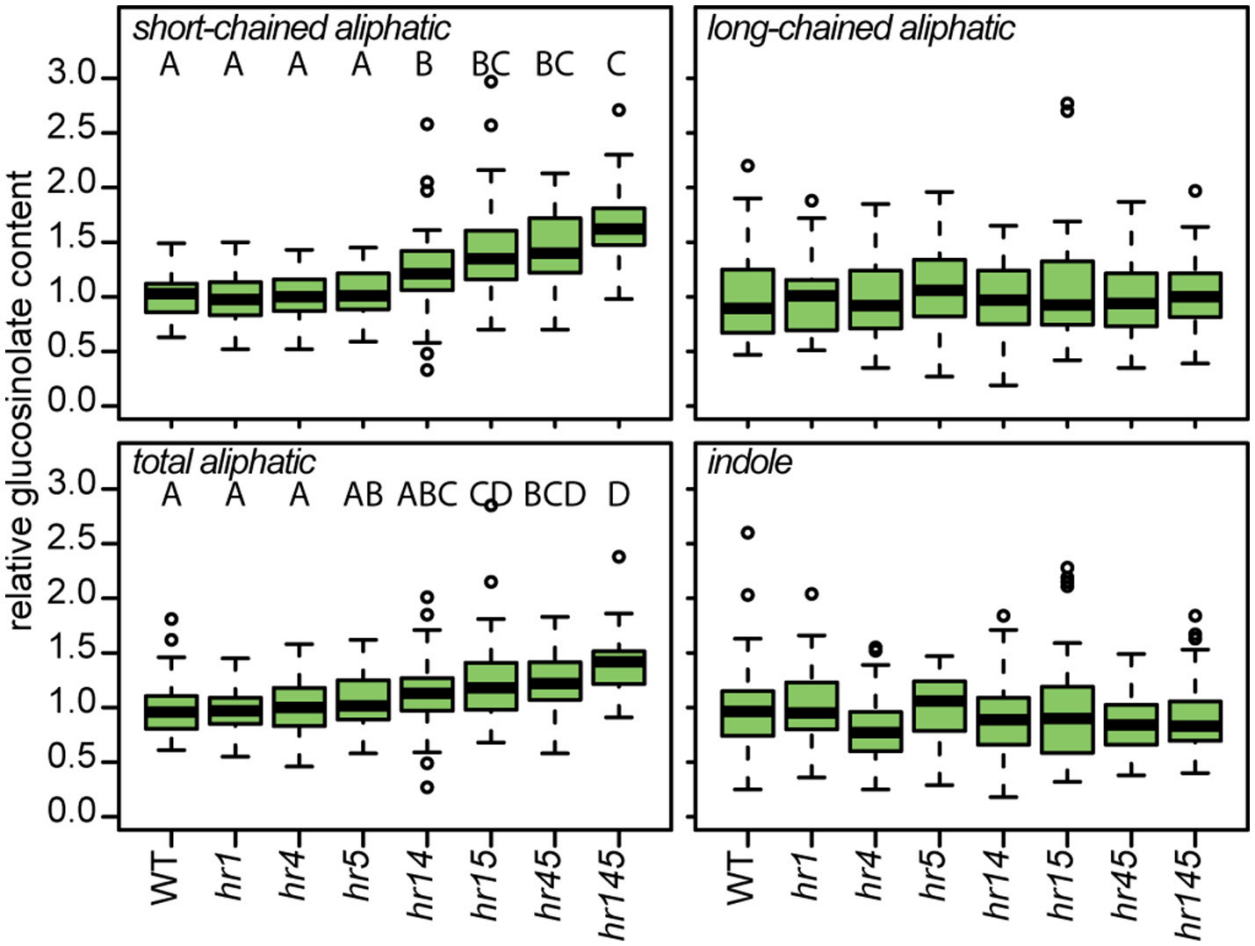
HR-like genes influence the accumulation of a specific class of glucosinolates. Relative content of different classes of glucosinolates in 12-day-old plants carrying mutant alleles of one or several *HR-like* genes [Two-way ANOVA (Table S5) followed by *post-hoc t*-test, Holm-adjustment for multiple testing, *p* < 0.05, *n* = 43–48].

We further investigated whether the identified HR-like proteins are functionally linked to glucosinolate accumulation by measuring glucosinolate concentrations in plants carrying mutations in one (*hr1, hr4, hr5*), two (*hr14, hr15, hr45*), or all three (*hr145*) of the genes (Figure 6). As observed before for *hr4* and *hr5*, the single mutant alleles did not result in a significant change in short-chained aliphatic, long-chained aliphatic, total aliphatic, indole or total glucosinolate content compared to wildtype. However, a specific and significant increase of 29–47% in short-chained aliphatic glucosinolates—i.e., glucosinolates derived from methionine with one, two or three additional methylene groups in the side chain—was observed in plants with two mutant alleles, and the triple insertion line *hr145* showed an increase of 66% in these compounds. The absence of an observable effect in the single mutants and the apparent additive effect of multiple insertions in genes of different members of this family suggest a redundant action of HR-like 1, 4, and 5 on the accumulation of short-chained aliphatic glucosinolates.

## Discussion

In this study, we set out to identify protein interactors of the aliphatic and indole glucosinolate biosynthetic pathways using the pathway-specific enzymes CYP83A1 and CYP83B1 as baits in untargeted yeast 2-hybrid and Co-IP approaches. Our results highlight the potential interaction of the CYP83s with a large number of proteins. Noticeably, neither biosynthetic enzymes from the pathways of the glucosinolate core structure nor any apparent scaffolding proteins or assembly chaperones were among the total of 75 (CYP83A1 bait) and 70 (CYP83B1 bait) putative interactors. Instead, our findings draw attention to the protein-protein interaction networks into which the biochemical pathways are embedded. Most interestingly, our results seem to indicate a link to innate immunity through interactions with proteins involved in cell death regulation and HR.

### Complementary approaches uncover diverse, comprehensive networks of interactors

When interpreting results, it is crucial to consider technical strengths, but also biases and limitations of the chosen methods. In contrast to Co-IP, yeast 2-hybrid can detect interactions with very lowly abundant proteins, provided the respective sequence is present and successfully taken up by yeast cells. However, the employed cDNA libraries do not contain full coverage of the transcriptome. The choice of library can be critical (Brückner et al., 2009), as also suggested by the little overlap between our screens of different libraries with identical baits. Furthermore, the probability of a prey protein being successfully expressed in yeast increases with abundance of respective plasmids. Long transcripts are more likely to be incompletely reverse transcribed and thus lead to incomplete or misfolded protein fragments. In this study, the yeast 2-hybrid approach showed an additional bias toward membrane-bound proteins, which might in part be explained by the usage of membrane-anchored baits. Together, this leads to a technical bias toward shorter, membrane-bound proteins, encoded by transcripts of high abundance.

Frequent presence amongst colonies—as observed with HR-like 5 in the yeast 2-hybrid screens—can also be linked to interaction strength, as stronger interactions result in more successful colony formation events. Available transcript data (Winter et al., 2007) for this gene shows lower or comparable levels to the lowly expressed *CYP83A1* and *CYP83B1* in relevant tissues, suggesting that the gene is not enriched in our libraries. The relatively large number of candidate interactors that, in addition to interacting with both CYP83A1 and CYP83B1, also interacted with LargeT suggests that some of the interactions are of promiscuous nature and appear between a broad range of proteins. Finally, in certain cases, interactions can involve the employed protein tags and thereby contribute to the relatively high false positive rate of yeast 2-hybrid screens reported in the literature (von Mering et al., 2002). Affinity enrichment methods such as Co-IP have the advantage that it is possible to detect indirect protein-protein interactions. Protein extraction itself, however, may disrupt unstable or otherwise transient protein complexes, particularly in the presence of detergents used to solubilize membrane proteins. This problem is further aggravated by the need of subsequent wash steps in order to reduce unspecific interactions of highly abundant proteins—often photosynthetic proteins in plant samples (Smaczniak et al., 2012; Gupta et al., 2015). Losses due to adsorption during sample preparation favor hydrophilic proteins, and tryptic digest followed by MS-based proteomics favors longer proteins, which further contributes to methodical bias toward stable interactions between soluble, abundant proteins.

A relatively large proportion of the candidates detected in Co-IP is predicted or has been determined to localize to plastids. It is important to note that predictions are not always correct and that even in cases where experimental evidence is available, the subcellular localization indicated in Tables 1–3 only reflects a consensus based on often conflicting findings. Fractions of protein populations can localize to different compartments, proteins could interact during protein translocation or be localized to the periphery of plastids where physical contact to ER membranes is possible. We thus decided not to disregard candidates based on their reported or predicted subcellular localization.

The question whether the observed interactions occur *in planta*, as well as their biological relevance and mode of action, can only be addressed on a case-by-case basis. When interpreting annotations and functional networks, it has to be considered that the underlying connectivity is often only based on predictions, and that large parts of the proteome are still unexplored. Many proteins lack proper annotation, as exemplified by the large group of unclassified proteins in our network analysis. It is estimated that as much as half of all eukaryotic proteins cannot be assigned a function or structure, and that especially shorter proteins belong to the so-called “dark proteome” with no assigned structural similarity to described proteins and generally little available knowledge about their functions (Perdigao et al., 2015).

In summary, the complementary strengths of the employed methods contribute to a high diversity of proteins found by our independent approaches, allowing for a more comprehensive view of the protein-protein interaction networks. At the same time, the sensitivity, the choice of experimental design as well as the inherent limitations and biases complicate comparisons to other experiments, which may explain the few previously reported interactors in our datasets.

### Network analysis allows interpretation of fragmentary data

Network analysis enables assessment of functional associations between proteins based on a variety of properties, including physical protein-protein interactions, co-expression, homology, and participation in pathways and biological processes (von Mering et al., 2005; Szklarczyk et al., 2015). In our network analysis, approximately two thirds of the candidates could be classified as belonging to one of three GO term enriched clusters. The proteins in cluster I (*organelle organization* and *vesicle-mediated transport*) were mainly identified by yeast 2-hybrid and contain putative interactors of CYP83A1. Cluster II consists of detected interactors of CYP83A1 as well, and almost exclusively of proteins identified in Co-IP experiments. The cluster contains proteins involved in the *generation of precursor metabolites and energy* and *porphyrin-containing compound metabolic process*. The interaction between a glucosinolate biosynthetic enzyme and these proteins is intriguing, as it could point to e.g. a feedback coupling of the pathway to the production of required precursors from primary metabolism. Cluster III is the only functionally enriched cluster of detected interactors of CYP83B1 and contains proteins involved in mRNA processing and splicing.

A possible explanation for the high number of membrane-associated proteins grouped into cluster I could be the spatial organization of glucosinolate pathways within the cell. The CYP83A1 enzyme localizes to the cytosolic face of the ER, while downstream enzymes of the pathways are soluble in the cytosol. Micro-compartments as formed by membrane invaginations or protein complexes at the membrane surface might fulfill a role in regulating local concentration of intermediates, and in effect promote substrate channeling and circumvent escape of instable or toxic intermediates, similar to what has been proposed for metabolons. Such micro-compartmentalization has been observed between the ER and plastids, where it allows for metabolic continuity between these compartments (Mehrshahi et al., 2013, 2014). Given that methionine chain-elongation steps in aliphatic glucosinolate biosynthesis are catalyzed by plastidic enzymes, a similar mechanism could facilitate this pathway by allowing for an elegant way of feeding chain-elongated methionine from the plastids into the ER-associated cytosolic aliphatic glucosinolate core pathway. Micro-compartmentalization of glucosinolate pathways could also reflect subpopulations of the biosynthetic machinery for specific purposes. For instance, the ER-associated CYP83A1 and CYP83B1 were both found enriched in Co-IP experiments with the late endosomal vesicle marker ARA7 (Heard et al., 2015). This localization of the CYP83 enzymes away from the ER membrane could be promoted by interacting proteins as found in cluster I of our network, and possibly serve biosynthesis and storage of glucosinolates destined for vesicular export. Indeed, such exosomes containing components of the glucosinolate-myrosinase defense system were identified and proposed as an apoplastic storage space for antimicrobial compounds or their precursor molecules (Rutter and Innes, 2016). Intriguingly, these exosomes were enriched in stress-associated proteins, and their secretion was stimulated upon *Pseudomonas syringae* infection, suggesting a role in immunity (Rutter and Innes, 2016). Another example of micro-compartmental localization of glucosinolate biosynthesis is the indole glucosinolate-modifying enzyme CYP81F2 in epidermal cells, which in challenged cells shows a reticulate distribution, as well as focal accumulation at the site of microbial attack (Fuchs et al., 2016).

VAMP722 and VAMP721 that both have an established role in immunity (Yun et al., 2013; Kim et al., 2014), were also found as interactors of CYP83A1 and contribute to cluster I in the present study. Cluster I furthermore contains the known interactor BI1, a regulator of cell death. While we did not detect this interactor in any of our screens, several of our candidate interactors connected with this protein in the network analysis: the mentioned VAMP722 (At2G33120), HA1 (At2G18960), CAM2 (At2G41110), and CB5-B (At2G32720). BI1 interacts physically with CB5-B and calmodulins (Ihara-Ohori et al., 2007; Kawai-Yamada et al., 2009), and these protein-protein interactions are proposed to enable BI1 to act on fatty acid modification and consequently fulfill its role in cell death suppression (Nagano et al., 2009). Both CB5-B and CAM2/AtCAL5—one of seven Arabidopsis calmodulins—were also found as interactors of CYP83A1 in our screens. The link of BI1 with CB5-B, CAM2, the fatty acid desaturase 2 (FAD2), as well as reduced oleate desaturation 1 (ROD1) is of particular interest, as lipid composition is a major cue in the onset of programmed cell death in response to stress (Liang et al., 2003; Nagano et al., 2009). The interplay between these proteins could explain the regulatory role of BI1, as lipid composition can be crucial for enzyme activity and complex formation in biochemical pathways (Laursen et al., 2016). An impact of lipid composition on glucosinolate metabolism, facilitated by CYP83A1's proximity to the respective protein complexes, seems plausible.

In conclusion, through network analysis, we were able to classify and group many of the detected interactors, and show that the interactors of CYP83A1 and CYP83B1—marker enzymes for aliphatic and indole glucosinolate biosynthesis—are functionally different, probably reflecting that aliphatic and indole glucosinolates function in different biological contexts.

### Linking glucosinolate metabolism with hypersensitive response/innate immunity

The network containing BI1 suggests a link from glucosinolate metabolism to cell death regulation. BI1 has been directly linked to regulation of HR (Matsumura et al., 2003; Kawai-Yamada et al., 2009), which is triggered by pathogen effectors and induces a rapid programmed cell death reaction at the site of infection, thereby counteracting the spread of biotrophic pathogens (Wu et al., 2014). It is known that indole glucosinolates are essential for HR-mediated immunity (Bednarek et al., 2009; Clay et al., 2009; Johansson et al., 2014) and that intermediates and products of aliphatic glucosinolate biosynthesis play an essential role in interactions between plants and the pathogenic fungus *Erysiphe cruciferarum* (Weis et al., 2014). Additionally, a role for glucosinolates and derived metabolites, as well as other specialized metabolites, has been proposed in regulation of innate immunity responses (Bednarek, 2012). Based on our findings, as well as the interactions described in previous studies, we propose that an interaction network between proteins involved in innate immunity and glucosinolate biosynthesis links these defense strategies.

As novel candidates for such a link, we investigated members of a small family of proteins containing a domain associated with HR. These HR-like proteins were detected as interactors of both CYP83 enzymes in this study, and are further linked to HR via yeast 2-hybrid interactions with established HR regulators (Arabidopsis Interactome Mapping Consortium, 2011). For example, both HR-like 5 and HR-like 1 interact with ANAC089 (associated with HR and ER stress-induced cell death, Yang et al., 2014) and VAP27 (an interactor of ACD11, a known regulator of HR, Petersen et al., 2009). HR-like proteins interact with a multitude of proteins and may—in concert with the many other glucosinolate pathway interactors—lead to an alteration in glucosinolate metabolism in response to a defense cue. This is demonstrated by our analysis of mutant alleles of three HR-like proteins. Plants with insertions in multiple *HR-like* genes showed an apparent additive increase in the accumulation of short-chained aliphatic glucosinolates.

Phenotypic analyses of genetic perturbations of candidate genes are a versatile approach to link genes to potential biological functions. In our sample of T-DNA insertion lines in genes of 34 proteins found to interact with either CYP83A1 or CYP83B1, a considerable proportion showed a trend, some a significant increase in aliphatic glucosinolate levels under our experimental conditions (Figure 3). It has been reported earlier that glucosinolate metabolism is generally sensitive to mutations of leaf-expressed genes (Chan et al., 2011). It can therefore be assumed that the observed impact on glucosinolate metabolism can partially be explained by this background influence, caused by any genetic perturbation that affects the plant's physiology. However, the correlation of interactors detected with CYP83A1 as bait and the changes in aliphatic glucosinolate accumulation are an indication that the detected protein-protein interactions indeed have an impact on glucosinolate accumulation. It is known that many glucosinolate phenotypes are dependent on environmental factors (Gigolashvili et al., 2008; Frerigmann and Gigolashvili, 2014; Li et al., 2014; Burow, 2016), and especially interactors related to defense signaling might only reveal condition-specific phenotypes. The overall trend in our high-throughput phenotypic analysis, along with the observations in plants carrying mutations in *HR-like* genes, point toward a role of protein-protein interactions as negative regulators of basal aliphatic glucosinolate levels. Perturbations of the interaction network upon attack (possibly mimicked by the genetic perturbations in T-DNA mutant lines) could give a rapid boost in glucosinolate production, even before the biosynthetic machinery has been upregulated.

Using complementary untargeted methods, we discovered a large number of novel candidate interactors of glucosinolate biosynthetic enzymes. Through network analysis, we identified networks of interacting proteins involved in biological processes that were clearly distinct between the marker of the aliphatic glucosinolate pathway, CYP83A1, and the marker of the indole glucosinolate pathway, CYP83B1. Our results suggest that—rather than forming a stable metabolon structure—the glucosinolate pathways may assemble stochastically through a multitude of transient interactions in a highly organized microenvironment. Based on the interaction networks, we propose a protein level link between innate immunity and glucosinolate metabolism. Future investigations will unravel the molecular link between HR-like proteins and aliphatic glucosinolates, as a functional link to HR seems plausible.

## Experimental procedures

### Bait cloning and evaluation

The coding sequences of CYP83A1 (At4G13770) and CYP83B1 (At4G31500) were PCR amplified using the Phusion U DNA polymerase (Thermo Scientific) and *A. thaliana*, ecotype Columbia-0 cDNA as template with primer pairs 83A1_Sfi_F/83A1_Sfi_nostop_R and 83A1_Sfi_F/83B1_Sfi_nostop_R, respectively. The PCR products as well as the pDHB1 bait vector (Dualsystems Biotech) were digested with SfiI (New England Biolabs) and the CYP83A1 and CYP83B1 coding sequences ligated to the vector backbone (Figure S7). The proper fusion and insertion of all sequences was confirmed by DNA sequencing. Oligonucleotide sequences are listed in Table S1.

*Saccharomyces cerevisiae* strain NMY51 (Dualsystems Biotech, MATa his3Δ200 trp1-901 leu2-3,112 ade2 LYS2::(lexAop)_4_-HIS3 ura3::(lexAop)_8_-lacZ ade2::(lexAop)_8_-ADE2 GAL4) was transformed with the pDHB1-CYP83A1 and pDHB1-CYP83B1 bait plasmids by the LiAc/PEG method (Gietz and Schiestl, 2007) and selected for the presence of the plasmid on synthetic complete dropout medium (Sunrise Science Products) with yeast nitrogen base (Duchefa) and glucose, lacking leucine (SD-L), generating the two bait carrying strains for the library screens. These strains were tested for bait functionality and background growth by transformation with the control preys pPR3-N (empty prey vector), NubI (positive control prey) and the unrelated prey ΔP53, interactor of the control bait LargeT (SV40 large T antigen) (Möckli et al., 2007). The selection stringency was titered by the addition of increasing concentrations 3-Amino-1,2,4-Triazole (3AT, Sigma) to SD media. 3AT is a competitive inhibitor of the *HIS3* reporter that increases the stringency of the histidine selection system and can be used to enable screens with moderately autoactivating bait proteins (Snider et al., 2010).

### Library construction

Two independent cDNA libraries based on the pPR3-N prey vector (Dualsystems Biotech) were generated from centers of rosettes of 3-week-old plants grown on soil and hypocotyls from 10-day-old seedlings grown on Murashige and Skoog medium including vitamins (M0222, Duchefa), respectively. The plant tissues were collected on ice and subsequently frozen in liquid nitrogen. Total RNA was extracted from 30 mg of hypocotyls and 60 mg of nodes using the Qiagen RNeasy plant mini kit. The obtained RNA was analyzed using an Agilent Bioanalyzer 2100. Double stranded cDNA was produced with Sfi-oligo-dT and Sfi-random primers using the Clontech SMART® cDNA library construction kit following the manufacturer's instructions. The cDNA and the empty pPR3-N vector were digested with SfiI, the linearized plasmid DNA was treated with calf intestine phosphatase (Sigma Aldrich) and purified by gel electrophoresis and the QIAquick Gel Extraction Kit (Qiagen). 100 ng of the SfiI digested and dephosphorylated pPR3-N vector was ligated with T4 DNA ligase (New England Biolabs) to 0.5, 1, or 1.5 μL of SfiI-digested cDNA. After incubation overnight, the ligation mixture was sodium acetate/ethanol precipitated and resuspended in 5 μL H_2_O. 25 μL of MegaX DH10B™ T1R Electrocomp™ Cells (Invitrogen) were used for transformation with 1 μL of ligation mix. After 1 h of recovery in recovery medium, dilutions of the transformation mixtures were plated on LB agar plates containing 100 μg/mL Ampicillin to determine the complexity of the libraries. The most complex libraries contained 3.8 × 10^5^ and 4.4 × 10^5^ transformants for the node- and hypocotyl library, respectively, and were selected for the untargeted yeast 2-hybrid screens. The node primary library was diluted to 40.6 mL and the hypocotyl primary library was diluted to 51.2 mL with LB medium. The cell suspensions were plated on 12 cm square LB agar plates containing 100 μg/mL Ampicillin in aliquots of 150 μL and the plates were incubated for 18–20 h at 37°C. Plasmids from 12 randomly chosen colonies for each library were extracted and digested with SfiI. Insert sizes of between ca. 600 bp and ca. 1,650 bp were observed for both libraries. All colonies were then resuspended in 3 mL of LB medium containing 25% glycerol per plate, scraped off the agar with a Drigalski spatula and combined to yield the node and hypocotyl libraries, respectively. Plasmids were isolated from 1.5 mL of each library using an alkaline lysis plasmid extraction protocol.

### Untargeted yeast 2-hybrid screens

Yeast cells containing the bait proteins were transformed with the prey libraries using the LiAc/PEG method (Gietz and Schiestl, 2007) and following the instructions of the Dualsystems Biotech DUALhunter kit manual. Dilutions of the library transformation mixtures were plated on SD-LW medium to determine the transformation efficiency (Table S2) and the remaining mixtures were plated on synthetic complete dropout medium lacking adenine, histidine, leucine and tryptophan (SD-AHLW) and containing 1 mM of 3AT in the case of CYP83A1 and incubated for up to 6 days at 30°C. Cells from growing colonies were allowed to grow in liquid SD-LW medium and the containing plasmids extracted and used for *E.coli* transformation. Prey plasmids were amplified in *E. coli*, extracted and sequenced to identify insert sequences. The sequences were analyzed by the basic local alignment search tool (BLAST) of The Arabidopsis Information Resource (TAIR, www.arabidopsis.org).

### Cloning of prey constructs for pairwise interaction analysis

For high throughput cloning of sequences into the pPR3-N prey vector, a USER™ (Nour-Eldin et al., 2010) compatible version of the vector was created by SfiI digestion and insertion of a USER™ cassette (oligo sequences USER_F and USER_R). The coding sequences of prey genes were amplified by specific primers including USER™ sites (Table S1) and inserted into the vector by USER™ cloning (Figure S7). All sequences were confirmed by DNA sequencing.

### Drop test

To confirm pairwise interactions, yeast cells were co-transformed with bait and prey plasmids by the LiAc/PEG method (Gietz and Schiestl, 2007) and selected for the presence of both plasmids on SD-LW medium. Cells from three individual colonies were grown overnight in liquid SD-LW medium, sedimented by centrifugation, resuspended in sterile water and brought to an OD_600_ of 0.5. Dilution series of 10^−1^–10^−4^ were prepared in sterile water and 5 μL of each dilution was dropped on plates containing SD-LW medium or SD-AHLW, containing 1 mM 3AT in the case of CYP83A1 baits. The plates were incubated for 3 days at 30°C before analyzing yeast growth.

### Glucosinolate analysis

The SALK T-DNA insertion lines analyzed in this study are listed in Table S3. Glucosinolates were extracted from leaves of 3-week-old plants cultivated in a randomized setup on soil in a growth chamber with constant long day light conditions (16 h light, 100–140 μE/(m^2^ s), 21°C, 70% relative humidity) as desulfo-glucosinolates. For the analysis of single, double and triple insertion lines in *HR-like* genes, plants were grown on vertical plates containing Murashige and Skoog medium including vitamins (M0222, Duchefa) under the same conditions for 12 days before desulfo-glucosinolate extraction. Desulfo-glucosinolates were then analyzed by UHPLC/TQ-MS on an Advance™-UHPLC/EVOQ™ Elite-TQ-MS instrument (Bruker) equipped with a C18 reversed phase column (Kinetex 1.7 μ XB-C18, 10 cm × 2.1 mm, 1.7 μm particle size, Phenomenex) as described previously (Jensen et al., 2015; Crocoll et al., 2016).

### Statistical analysis

We used R version 3.2.0 (2015-04-16) for statistical analysis (R Core Team, 2015). We tested the *A. thaliana* Columbia-0 wildtype and the different transgenic lines with the ANOVA function for the following linear model for effects on total indole or aliphatic glucosinolates separately: Indole glucosinolates or aliphatic glucosinolates = genotype + tray (Table S4). Specific differences between genotypes and the wildtype were tested *post-hoc* using the pairwise.t.test function with a Holm-adjustment for multiple testing. For the analysis of insertions in *HR-like* genes, glucosinolate contents were normalized to corresponding wildtype levels and tested with the following linear model: Aliphatic, short- or long-chained aliphatic or indole glucosinolates = genotype + experiment (Table S5). Specific differences between genotypes were tested *post-hoc* using the pairwise.t.test function with a Holm-adjustment for multiple testing.

### Functional enrichment of gene ontology categories

Enrichment of functional categories was performed using PANTHER (version 11.1) (Mi et al., 2017) with the *A. thaliana* PANTHER GO-Slim biological process annotation released 2016-10-24. Unless specified otherwise, the whole genome list was used as reference list and, where possible, multiple testing correction was done with Bonferroni. GO terms with *p* < 0.05 were considered as over-represented.

### Sequence alignment and phylogenetic tree construction

Coding sequences of the six HR-like proteins At5G43460, At4G14420, At1G04340, At3G23175, At3G23180, and At3G23190 were retrieved from The Arabidopsis Information Resource (TAIR, www.arabidopsis.org) and aligned using the create alignment function of CLC Main Workbench (Version 7.6.1, Quiagen Aarhus A/S). The identity at the amino acid level was obtained by aligning the corresponding protein sequences and the pairwise comparison function of the program. The coding sequences were aligned using the ClustalW algorithm in JalView2 (Waterhouse et al., 2009) and a phylogenetic tree was generated using percentage identity from the Gblocks processed alignment.

### Construction and stable expression of fluorophore-tagged proteins in arabidopsis

To introduce the coding sequence of mVenus (Nagai et al., 2002) into the pCambia1300U plant expression vector (Nour-Eldin et al., 2006), the sequence was amplified with primer pair USER_XFP_F/USER_XFP_R, the forward primer containing the sequence of a USER™ cassette in front of the 5′ end of the coding sequence of the fluorescent protein. The PCR product was then introduced into the PacI and Nt.BbvCI digested pCambia1300U vector by USER™ cloning. The promoter sequences including upstream genomic sequences and the 5′ UTR of CYP83A1 and CYP83B1 (1,033 and 1,001 bp upstream of the start codon, respectively) were amplified from genomic DNA of *A. thaliana* ecotype Columbia-0 with the primer pairs Pro_83A1_F/Fus_Pro_83A1_R and Pro_83B1_F/Fus_Pro_83BA1_R. The corresponding coding sequences were amplified from *A. thaliana* ecotype Columbia-0 cDNA with primer pairs Fus_83A1_F/83A1_Nostop_R and Fus_83B1_F/83B1_Nostop_R. The promoter and coding sequences were fused by USER™ fusion (Geu-Flores et al., 2007) and inserted into the USER™ cassette of the opened pCambia1300-mVenus vector. The proper fusion and insertion of sequences was confirmed by DNA sequencing. Oligonucleotide sequences are listed in Table S1. The plasmids were introduced into *Agrobacterium tumefaciens* strain GV3101 and *A. thaliana* ecotype Columbia-0 was transformed by the floral dip method (Clough and Bent, 1998). Transgenic plants were selected by germination on 1% agar plates (½ Murashige and Skoog medium including vitamins, M0222, Duchefa, containing 100 μg/mL Hygromycin, H0192.0001, Duchefa) and cultivated in a growth chamber with constant long day light conditions (16 h light, 100–140 μE/(m^2^ s), 21°C, 70% relative humidity). For each construct, 5 individual transgenic lines were analyzed for their global expression pattern by fluorescence microscopy. No differences in expression patterns were observed and one representative line was chosen for the following work. The same transgenic plant lines carrying PrCYP83A1:CYP83A1-mVenus or PrCYP83B1:CYP83B1-mVenus were also used in Xu et al. (2016).

### Construction and transient expression of fluorophore-tagged proteins in tobacco

For the expression of the CYP83A1, CYP83B1, HR-like 1, 4, and 5 coding sequences in *Nicotiana benthamiana*, constructs were generated as follows. A plant expression cassette containing a ubiquitin (UBQ10) promoter and a RBC terminator were PCR amplified and inserted into the pEAQ-HT (Sainsbury et al., 2009) vector backbone. The coding sequences of mTurquoise2 (Goedhart et al., 2012) or mVenus were inserted into the resulting vector (Andersen et al., 2016). The respective coding sequences were PCR amplified with primer pairs CYP83A1_FW/CYP83A1_NSR, CYP83B1_FW/CYP83B1_NSR, At1G04340_FW/At1G04340_NSR, At4G14420_FW/At4G14420_NSR, and At5G43460_FW/At5G43460_NSR (Table S1) and inserted into the USER™ cassette of the fluorescent protein tagging vectors. All sequences were verified by DNA sequencing. *Agrobacterium tumefaciens* strain GV3101 was transformed with plasmids carrying the sequences of fluorophore-tagged fusion proteins and grown overnight at 28°C and 220 rpm in YEP medium containing the appropriate antibiotics. Cells were sedimented by centrifugation and resuspended in infiltration buffer (10 mM MES, 10 mM MgCl_2_ and 100 μM acetosyringone (3,5-Dimethoxy-4-Hydroxyacetophenone, Sigma-Aldrich), pH 5.6). After 2 h incubation at room temperature and 150 rpm agition, the cell suspensions were brought to an OD_600_ of 0.05 with infiltration buffer and infiltrated into the abaxial air space of leaves of *Nicotiana benthamiana* plants grown in small pots of 5.5 cm diameter in a green house at 24°C (day) and 18°C (night) with 50–60% humidity for approximately 3–4 weeks (to 4–6 leaves stage).

### Microscopy

Forty-eight hour after infiltration, confocal laser scanning microscopy was carried out on *Nicotiana benthamiana* leaves on a Leica SP5-X confocal microscope equipped with a HCX PL APO CS 63.0x1.20 WATER (1.2 numerical aperture, 63X magnification) objective. mVenus was excited at 514 nm and emission was collected at 524–550 nm. mTurquoise2 was excited at 458 nm and emission was detected at 468–518 nm. Chlorophyll autofluorescence was collected at 650–750 nm.

### Plant growth conditions for pulldowns

Plants of the transgenic Arabidopsis lines expressing PrCYP83A1:CYP83A1-mVenus or PrCYP83B1:CYP83B1-mVenus were grown on horizontal 1% agar plates (½ Murashige and Skoog medium including vitamins, M0222, Duchefa, containing 100 μg/mL Hygromycin, H0192.0001, Duchefa), alongside wildtype (Columbia-0) plants (no selection). Seeds were cold-stratified for 3 days before growing for 14 days in climate chambers with constant long day light conditions (16 h light, 100–140 μE/(m^2^ s), 21°C, 70% relative humidity).

### Co-immunoprecipitation

For each of the three independent experiments, three replicates of the three genotypes (wildtype, PrCYP83A1:CYP83A1-mVenus and PrCYP83B1:CYP83B1-mVenus) were prepared by harvesting 2–4 g seedlings per replicate. These seedlings were flash frozen in liquid nitrogen and ground using mortar and pestle. The resulting powder was mixed (1:1 w/v) with extraction buffer [150 mM Tris pH 7.5, 1 mM EDTA, 150 mM NaCl, 10% glycerol, 10 mM DTT, 1% IGEPAL CA630 (18896, Sigma-Aldrich), 2x Protease Inhibitor (11 873 580, Roche)]. After thorough mixing, the samples were incubated 30 min, shaking at 4°C. Samples were then spun at 13,000 g for 20 min at 4°C. The resulting supernatants were mixed with 20 μL of GFPtrap magnetic beads (Chromotek), which had been pre-equilibrated in extraction buffer. The samples were then incubated overnight at 4°C, with “end-over-end” rotation. Magnetic separation of beads and supernatant were carried out on ice and the supernatants were discarded, before performing 3 washes with 400 μL wash buffer each (150 mM NaCl, 50 mM TrisHCl, pH 8.0, 0.25% IGEPAL CA630). Samples were eluted using 30 μL 4% (w/v) SDS, 100 mM Tris/HCl pH 8.2, 0.1 M DTT) and heating to 95°C for 5 min before magnetic separation of beads and elution.

### Protein digest

Each individual elution was mixed with 200 μL UA buffer (8 M urea in 100 mM TrisHCl, pH 8.2) before being loaded onto a centrifugal filter unit (Microcon-30 kDa Centrifugal Filter Unit Ultracel-30 membrane, Millipore MRCF0R030) and then spun at 14,000 g for 20 min at room temperature. The flow-through was discarded and 200 μL of UA buffer were added to wash the filter by centrifugation at the above mentioned settings. Then, 100 μL of 0.05 M iodoacetamide (in UA buffer) were applied and incubated for 5 min before centrifugation. The filters were washed three times with 100 μL UA buffer and twice with 100 μL of 0.5 M NaCl. Each filter was then moved to a new collection tube and 120 μL 50 mM ammonium bicarbonate with 3 μg trypsin (Promega, V5073) were added to the filters, which were then shaken at 600 rpm for 1 min. The filters were incubated overnight at room temperature and the flow-through was eluted by centrifugation at 14,000 g for 20 min. Flow-throughs were acidified (pH < 2.5) using 10 % trifluoroacetic acid. Samples were then diluted in buffer S-A (2% acetonitrile, 0.1% formic acid) up to 1.5 mL and spun at 20,000 g for 15 min. The supernatants were each applied to a Sep-Pak column (Waters, WAT023590), pre-equilibrated using first 1 mL of buffer S-B (65% acetonitrile, 0.1% formic acid) and then 1 mL of buffer S-A. Three washes with 1 mL of buffer S-A were carried out before eluting using two applications of 0.5 mL buffer S-B. The eluted samples were completely dried in a vacuum centrifuge, after which they were stored at −20°C until further analysis.

### Mass spectrometric measurements

Mass spectrometry measurements were performed on a LTQ OrbiTrap Velos mass spectrometer (Thermo Fisher) coupled to a NanoLC-ultra (Eksigent) using electrospray ionization. A 15-cm capillary column, which was heated to 50°C and packed with 15 cm C18 beads with a diameter of 3 μm and a pore size of 100 Å was used for LC separation. Peptides were loaded on the column with a flow rate of 300 nL/min for 20 min and eluted with a flow rate of 300 nL/min for 65 min by an increasing gradient from 3% acetonitrile to 50% acetonitrile. The FT OrbiTrap was used for obtaining full scans at a range of 300–1,700 mass/charge, followed by MS/MS scans of the 20 highest parent ions. Dynamic exclusion was enabled at a duration of 45 s.

### Interpretation of MS/MS spectra

The obtained raw spectra were transformed to mgf data format and searched against the TAIR10 database (download on January 17th, 2011; Lamesch et al., 2012) with concatenated decoy database and supplemented with common contaminants (71,032 entries) using the Mascot algorithm (version 2.3.02) (Mascot Science). The search parameters used were: mass = monoisotopic, requirement for tryptic ends, 2 missed cleavages allowed, precursor ion tolerance = ±10 ppm, fragment ion tolerance = ±0.5 Da, variable oxidation of methionine (M, PSI-MOD name: oxidation, mono Δ = 15.995), protein N-terminal acetylation (protein N-term, PSI-MS name: acetyl, mono Δ = 42.010565 Da) and conversion of N-terminal glutamine into pyroglutamic acid (N-term Q, PSI-MS name: Gln → pyro-Glu, mono Δ = −17.026549 Da), and static carbamidomethylation of cysteine (C, PSI-MS name: carbamidomethyl, mono Δ = 57.0215) (Svozil and Baerenfaller, 2017). Peptide spectrum assignments with a Mascot score higher than the ion score that indicates identity or extensive homology (*p* < 0.05) in the respective sample were loaded into the pep2pro database. Peptides matching to known contaminants or to several proteins were excluded from further analyses, unless they belong to a different splice variant of the same protein or to a different locus, which shares exactly the same amino acid sequence (Baerenfaller et al., 2011; Hirsch-Hoffmann et al., 2012). For calculating the false discovery rate (FDR) the number of reverse hits was divided by the number of forward hits times 100%. All mass spectrometry proteomics data have been deposited to the ProteomeXchange Consortium (www.proteomexchange.org) via the PRIDE partner repository (Vizcaino et al., 2013) with the dataset identifiers PXD005585 and 10.6019/PXD005585. Furthermore, the data can be found in the pep2pro database at www.pep2pro.ethz.ch. Proteins were quantified by spectral counting and only proteins identified with at least five spectra were taken into consideration. Enriched proteins had to have at least 5 times more spectrum counts in the respective GFPtrap line compared to the wildtype control.

### Bioinformatics resources and analysis of interaction candidates

Descriptions and predicted transmembrane domains of the identified proteins (from both yeast 2-hybrid and Co-IP) were retrieved from The Arabidopsis Information Resource (TAIR, www.arabidopsis.org), via the bulk retrieval function. Molecular weights, amino acid lengths, isoelectric points, GRAVY scores and subcellular localization were retrieved from SUBA3 (Hooper et al., 2014) and the UniProt databases (Bateman et al., 2015; Boutet et al., 2016). Published interactions of CYP83A1 (At4G13770) and CYP83B1 (At4G31500) were extracted from the BioGrid database (Chatr-Aryamontri et al., 2015) (Table S6).

Our interaction network was constructed using the STRING database (Szklarczyk et al., 2015). AGI codes of all identified candidates—from yeast 2-hybrid, Co-IP and BioGrid—except UBQ3 (At5G03240), which was excluded because of its ubiquitous interactions, were queried in the STRING database, using a threshold score of 0.4. The resulting network was arranged and color-coded using the cytoscape software tool (Shannon et al., 2003).

## Author contributions

SN generated cDNA libraries and yeast 2-hybrid baits, planned and carried out yeast 2-hybrid experiments, generated Arabidopsis lines stably expressing fluorophore-tagged enzymes, generated lines carrying multiple T-DNA insertions, analyzed glucosinolate data, carried out statistical analysis and confocal microscopy and drafted the manuscript. DV planned, carried out and analyzed Co-IP experiments, carried out GO-term and network analysis and analyzed protein properties. JS carried out and analyzed Co-IP experiments. MBa cloned plasmids and carried out transient expression in *Nicotiana benthamiana*. KB carried out GO-term analysis and analyzed published transcript abundances. MBu generated and analyzed glucosinolate data and contributed to statistics, experimental and project design. BH was involved in experiment planning. All authors commented on the manuscript.

### Conflict of interest statement

The authors declare that the research was conducted in the absence of any commercial or financial relationships that could be construed as a potential conflict of interest.
