# Supplementary material for: Unravelling Protein-Protein Interaction Networks Linked to Aliphatic and Indole Glucosinolate Biosynthetic Pathways in Arabidopsis
Source: Front Plant Sci. 2017 Nov 29;8:2028. doi: 10.3389/fpls.2017.02028 (PMC5712850; doi:10.3389/fpls.2017.02028)
Supplement: Supplementary file 1 [file Image1.pdf]

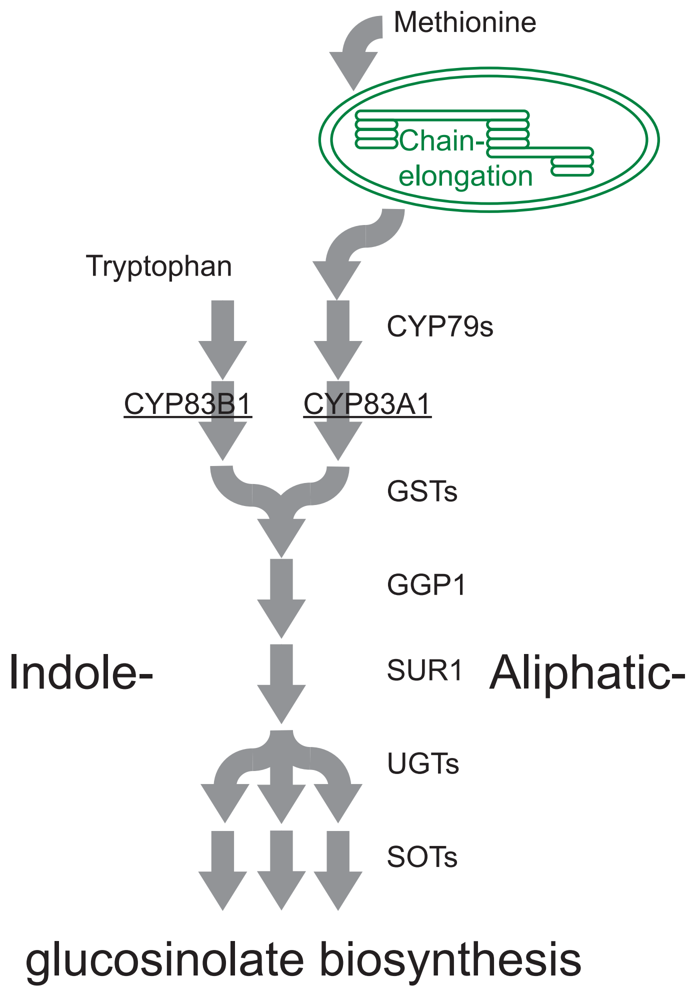

Figure S1: Overview of the aliphatic and indole glucosinolate biosynthetic pathways. The pathways consist of enzymes specific for either class of glucosinolates as well as common enzymes. Only aliphatic glucosinolate biosynthesis requires chain-elongation steps catalyzed inside plastids.

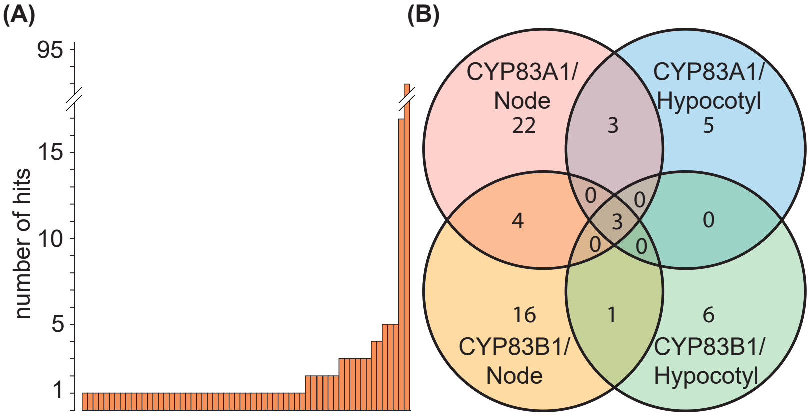

Figure S2: Frequency of identification of 60 candidate interactors identified by yeast 2-hybrid screens (**A**) and Venn diagram illustrating the overlap between results of the four individual screens with CYP83A1 and CYP83B1 baits and prey libraries generated from node or hypocotyl tissues (**B**).

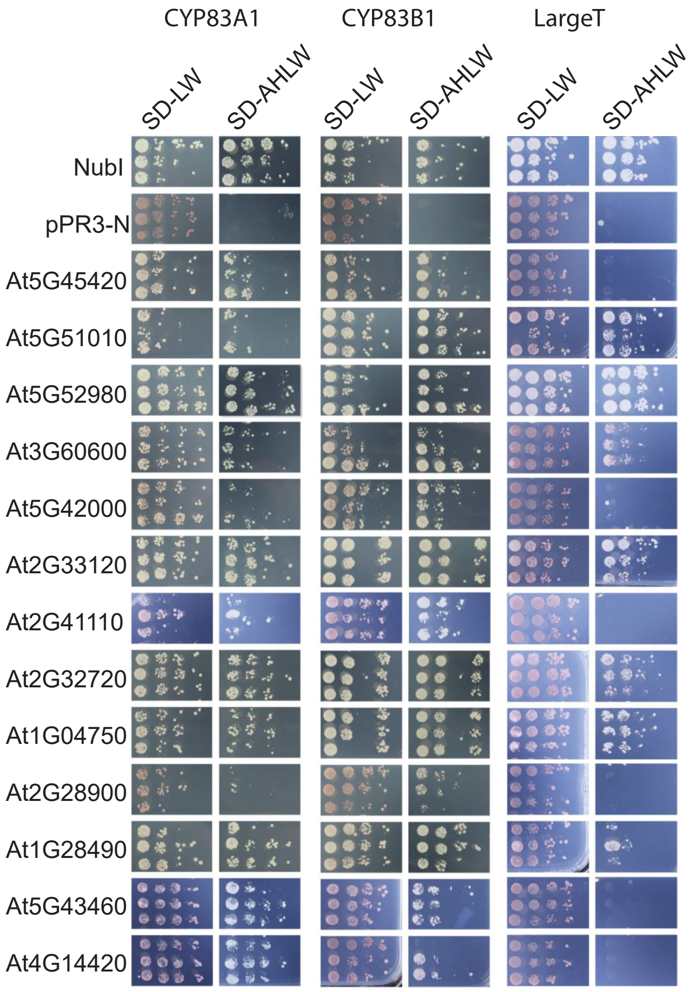

Figure S3: Interaction assay of interactors detected in yeast 2-hybrid screens with bait proteins CYP83A1, CYP83B1 and the unrelated control bait protein LargeT by pairwise co-expression in yeast. Cell suspensions were spotted on SD-LW (growth control) or SD-AHLW (selective medium for interacting protein pairs) medium in dilution series. NubI: positive control; pPR3-N: empty prey vector, negative control.

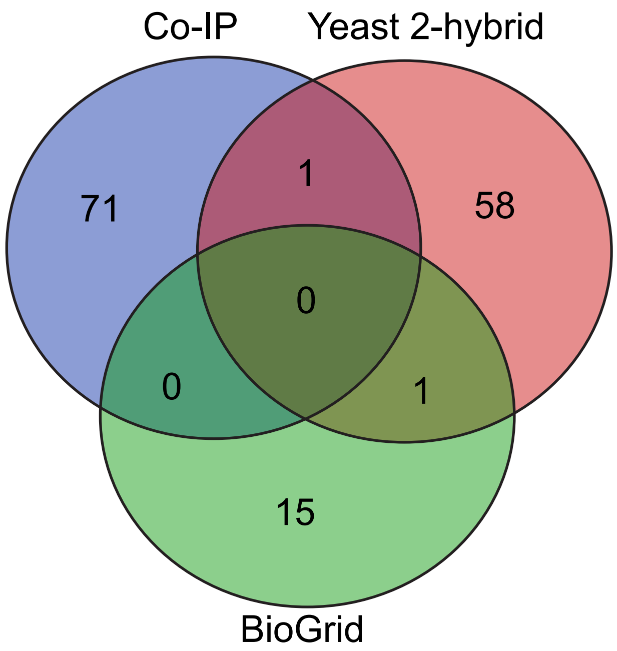

Figure S4: Venn diagram with the overlap between candidate interactor lists obtained by yeast 2-hybrid, Co-IP and from the BioGrid database of previously reported interactions.

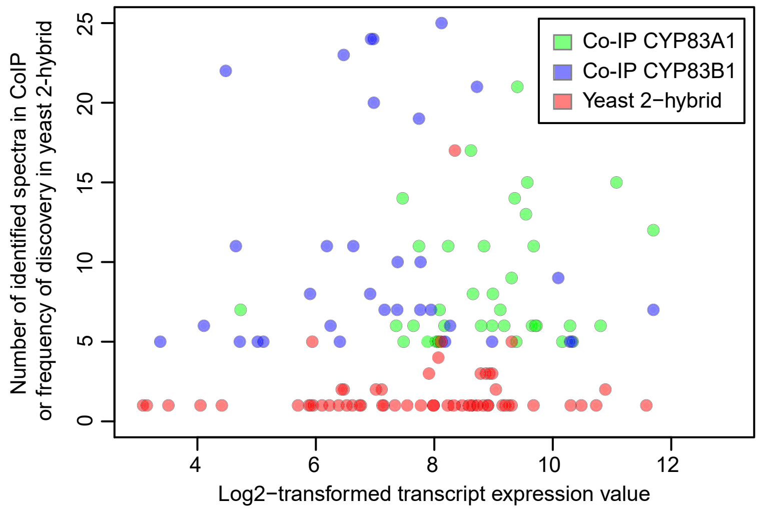

Figure S5: Number of identifications in yeast 2-hybrid screens and identified spectra in Co-IPs plotted against log2-transformed average transcript expression values as a proxy for protein abundance levels. Average expression values of the respective transcripts in vegetative rosettes were retrieved from the eFP anatomy browser of the BAR database (Winter et al., 2007).

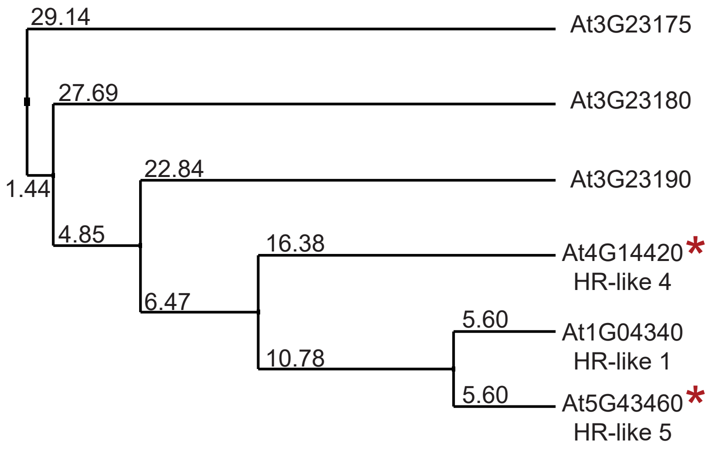

Figure S6: Phylogeny of six proteins annotated as HR-like lesion-inducing protein-related protein. Numbers represent distances based on neighbor joining by percentage identity. Proteins identified as interacting with glucosinolate biosynthetic proteins in yeast 2- hybrid screens are labeled with *.

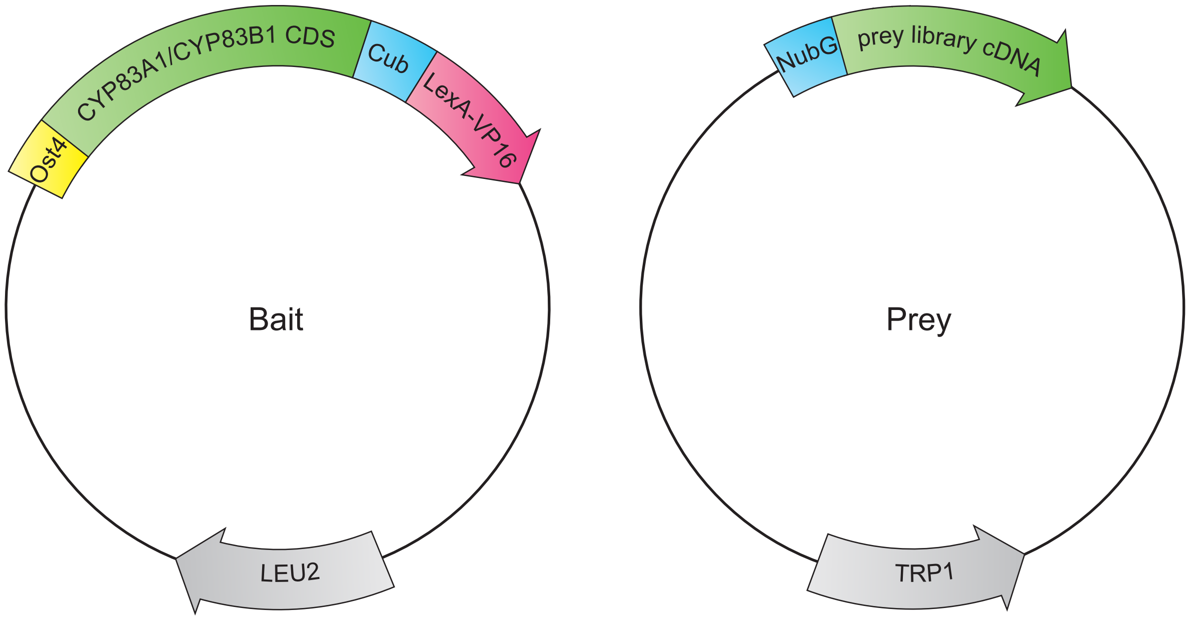

Figure S7: Schematic overview of bait and prey constructs used in yeast 2-hybrid screens. The bait vector encodes a fusion protein containing the Ost4-sequence to ensure correct integration into the yeast ER membrane, followed by the coding sequence (CDS) of either CYP83A1 or CYP83B1, the C-terminal half of ubiquitin (Cub) and the transcriptional activator LexA-VP16. The prey constructs contain the cDNA libraries generated from Arabidopsis mRNA and the sequence of a mutagenized N-terminal half of ubiquitin (NubG). The mutation prevents the ubiquitin halves from interacting intrinsically. The plasmids contain yeast selection markers (LEU2 and TRP1).

Upon protein-protein interaction, NubG and Cub reconstitute a functional ubiquitin, leading to proteolytic cleavage and release of LexA-VP16, and thereby the activation of the reporter system.

Table S1: Oligonucleotide sequences

83A1_Sfi_F	ATTGAGGCCATTACGGCCATGGAAGATATCATCATCGGCG
83B1_Sfi_F	ATTGAGGCCATTACGGCCATGGATCTCTTATTGATTATAGC
83A1_Sfi_nostop_R	TCAATGGCCGAGGCGGCCCAATACTTGTTCACTTTGTCTGG
83B1_Sfi_nostop_R	TCAATGGCCGAGGCGGCCCAGATGTGTTTCGTTGGTGCAAGAAC
USER_F	GGGGCTGAGGTTTAATTAAGGATCCTTAATTAAGCCTCAGCCCAAAAT
USER_R	GGGCTGAGGTTTAATTAAGGATCCTTAATTAAGCCTCAGCCCAAT
AT5G45420_FW	GGCTTAAUATGGATTTTTTCGACGAAGACAGG
AT5G51010_FW	GGCTTAAUATGGCGATGCAGCTTCC
AT5G52980_FW	GGCTTAAUATGGATCAACCCGACCCG
AT3G60600_FW	GGCTTAAUATGAGTAACATCGATCTGATTGGGA
AT5G42000_FW	GGCTTAAUATGTACGTAAGAGCACTTCCGA
AT2G33120_FW	GGCTTAAUATGGCGCAACAATCGTTGAT
AT2G41110_FW	GGCTTAAUATGGCGGATCAGCTCACA
AT5G61790_FW	GGCTTAAUATGAGACAACGGCAACTATTTTCC
AT2G32720_FW	GGCTTAAUATGGGAGACGAAGCAAAGATC
AT1G04750_FW	GGCTTAAUATGGCGCAACAATCGTTGA
AT2G28900_FW	GGCTTAAUATGCCTTCAAGCACATTCTCC
AT1G28490_FW	GGCTTAAUATGTCTTCAGCTCAAGATCCATTC
AT4G14420_FW	GGCTTAAUATGGAGTTAGCTTCTTTCCT
AT5G43460_FW	GGCTTAAUATGGGTTTCTTCTCTTTTCTTG
AT5G45420_REV	GGTTTAAUTTAATTAGCTGGAGTTTTCGAGCTC
AT5G51010_REV	GGTTTAAUTTAATTTCATGAGGTGCTATTGACATAGAAG
AT5G52980_REV	GGTTTAAUTTAATTCTATTGATTTTTCTTAGTAGTGGGAGTGA
AT3G60600_REV	GGTTTAAUTTAATTTTATGTCCTCTTCATAATGTATCCCAAAATT
AT5G42000_REV	GGTTTAAUTTAATTTCATTGGTCTCCATTGATTCCAAAT
AT2G33120_REV	GGTTTAAUTTAATTTTATTTACCGCAGTTGAATCCCC
AT2G41110_REV	GGTTTAAUTTAATTTCACTTAGCCATCATAACCTTCAC
AT5G61790_REV	GGTTTAAUTTAATTCTAATTATCACGTCTCGGTTGCC
AT2G32720_REV	GGTTTAAUTTAATTCTACCCTGATTTGGTGTAGATACG
AT1G04750_REV	GGTTTAAUTTAATTTTAACACTTAAACCCATGGCAAACT
AT2G28900_REV	GGTTTAAUTTAATTTCAGTAGAAATAATGATTGTTAACGAACTGA
AT1G28490_REV	GGTTTAAUTTAATTTTAGGTCAAGAAGACAAGAACGAATAG
AT4G14420_REV	GGTTTTAAUCTAGGCAGCAGCTTTCGGGTTA
AT5G43460_REV	GGTTTTAAUTCAGTTTGCTTTTCCCTTT
USER_XFP_F	GGCTTAAUTAAGGATCCTTAATTAAACCTCAGCACGTGAGCAAGGGCGAGGAGCTG
USER_XFP_R	GGTTTAAUTTACTTGTACAGCTCGTCC
Pro_83A1_F	GGCTTAAUCTGTAAGAGAAACGAAATTCGG
Fus_Pro_83A1_R	ATTCTTAGTUGTTACTACTTTTGAGTGTTACATTG
Pro_83B1_F	GGCTTAAUAAGTTGGAGGAAGATTTGACAG
Fus_Pro_83B1_R	ATTTTTTTCUGTTTGACTTACTTAAGTCTTTG
Fus_83A1_F	AACTAAGAAUGGAAGATATCATCATCGG
83A1_Nostop_R	GGTTTAAUATACTTGTTCACTTTCTCTGG
Fus_83B1_F	AGAAAAAAAUGGATCTCTTATTGATTATAGC
83B1_Nostop_R	GGTTTAAUGATGTGTTTCGTTGGTGCAAG
CYP83A1_FW	GGCTTAAUATGGAAGATATCATCATCGGCGTGG
CYP83A1_NSR	GGTTTAAUGGATACTTGTTCACTTTCTCTGGAACA
CYP83B1_FW	GGCTTAAUATGGATCTCTTATTGATTATAGCCGG
CYP83B1_NSR	GGTTTAAUGGGATGTGTTTCGTTGGTGCAAG
At1G04340_FW	GGCTTAAUATGGGTTTCTTCTCTTTTCTTG
At1G04340_NSR	GGTTTAAUCCAGCAGCTTTAGGCTTGGGAG
At4G14420_FW	GGCTTAAUATGGAGTTAGCTTCTTTCCT
At4G14420_NSR	GGTTTAAUGGGTTTGCTTTTGCCTTTGGAG
At5G43460_FW	GGCTTAAUATGGGTTTCTTCTCTTTTCTTG
At5G43460_NSR	GGTTTAAUCCGGCAGCTTTCGGCTTAGGTG

Table S2: Calculation of library transformation efficiencies. Dilutions of the four library transformations were plated on SD-LW medium and colony forming units were counted to determine the absolute number of transformants.

**Bait**<br>**Library**	**CYP83A1 Hypocotyl**	**CYP83A1 Node**	**CYP83B1 Hypocotyl**	**CYP83B1 Node**
**1:100**	50	164	58	142
**1:1000**	5		6	9
**1:10000**	1	1	2	2
**Titer**	2.5×10^5	8.2×10^5	2.9×10^5	7.1×10^5

Table S3: T-DNA insertion lines used to assess impact of putative interactors on glucosinolate accumulation

Gene	Insertion line	Name/Annotation
At1G04340	SALK_062978C	HR-like lesion-inducing protein-related
At1G04750	SALK_037273C	Vesicle associated membrane protein 721 (VAMP721); VAMP7B
At1G15500	SALK_031126C	NTT2; ATP:ADP antiporter
At1G16890	SALK_001977C	Ubiquitin-conjugating enzyme 36 (UBC36)
At1G26670	SALK_116968C	Vesicle transport V-SNARE 12 (VTI12); VTI1B
At1G28250	SALK_081490C	unknown
At1G28490	SALK_107167C	SYNTAXIN OF PLANTS 61 (SYP61); OSM1
At1G30935	SALK_084201C	F-box protein family pseudogene
At1G44920	SALK_137362C	unknown
At1G52870	SALK_133672C	Mpv17/PMP22 family; Peroxisomal membrane protein
At1G57765	SALK_103508C	unknown
At1G59890	SALK_043513C	SIN3-LIKE 5, SNL5
At1G66240	SALK_021013C	Homolog of anti-oxidant 1 (ATX1)
At2G05630	SALK_145672C	ATG8D
At2G32380	SALK_079608C	Transmembrane protein 97
At2G32720	SALK_009185C	Cytochrome B5-B (CB5-B)
At2G33120	SALK_103189C	Vesicle associated membrane protein 722 (VAMP722); Synaprobrevin-related protein 1 (SAR1)
At2G41110	SALK_089283C	CALMODULIN 2 (CAM2); ATCAL5
At3G48990	SALK_057339C	Acyl-activating enzyme 3 (AAE3)
At3G50685	SALK_133139C	unknown
At3G60600	SALK_021679C	VAMP/Synaprobrevin-associated protein 27-1 (VAP27, VAP27-1), Vesicle associated protein
At4G14420	SALK_008498C	HR-like lesion-inducing protein-related
At4G24920	SALK_021134C	Sec61 subcomplex, putative γ subunit; P-P-bond-hydrolysis-driven protein transmembrane transporter
At4G27500	SALK_009375C	Proton pump interactor 1 (PPI1)
At4G30950	SALK_000835C	Fatty acid desaturase 6 (FAD6); FADC; SFD4; Stearoyl desaturase defficiency 4
At5G16830	SALK_054274C	Syntaxin of plants 21 (SYP21); PEP12
At5G20130	SALK_058047C	unknown
At5G24680	SALK_051987C	Peptidase C78, ubiquitin fold modifier-specific peptidase 1/ 2
At5G42570	SALK_142314C	B-cell receptor-associated 31-like
At5G43460	SALK_030116C	HR-like lesion-inducing protein-related
At5G43580	SALK_057639C	Unusual serine protease inhibitor (UPI)
At5G52240	SALK_035827C	Membrane-associated progesterone binding protein 5 (MAPR5); ATMP1; Membrane steroid binding protein 1 (MSBP1)
At5G60920	SALK_106466C	COBRA (COB)
At5G61790	SALK_083600C	Calnexin 1 (CNX1)
At5G67600	SALK_038916C	WIH1 (WINDHOSE 1)

Table S4: Two-way ANOVA testing for effects of genotype and plant tray on aliphatic and indole glucosinolates with the linear model aliphatic or indole glucosinolates = Genotype + Tray.

		Df	Sum Sq	Mean Sq	F value	Pr(>F)
Aliphatic glucosinolates	Genotype	34	1782	0.05241	4038	1.17e-11***
	Tray	1	0.001	0.00109	0.084	0.772
	Residuals	348	4517	0.01298		
Indole glucosinolates	Genotype	34	0.1516	0.004460	1100	0.326
	Tray	1	0.0005	0.000518	0.128	0.721
	Residuals	348	14106	0.004053		

*** indicates $p < 0.001$

Analyzed genotypes are wildtype (n = 22) or T-DNA insertion lines (n = 6-12) listed in Table S3.

Aliphatic glucosinolates:

3-methylthiopropyl-, 3-methylsulfinyl-, 4-methylthiobutyl-, 4-methylsulfinylbutyl-, 5-methylsulfinylpentyl-, 7-methylthioheptyl-, 7-methylsulfinylheptyl-, 8-methylthiooctyl-, 8-methylsulfinyloctyl-glucosinolate

Indole glucosinolates:

Indol-3-ylmethyl-, 4-methoxy-indol-3-ylmethyl-, N-methoxy-indol-3-ylmethyl-glucosinolate

Table S5: Two-way ANOVA testing for effects of T-DNA insertions on different classes of glucosinolates with the linear model short-chained aliphatic, long-chained aliphatic, total aliphatic or indole glucosinolates = Genotype + Experiment.

		Df	Sum Sq	Mean Sq	F value	Pr(>F)
Short-chained	Genotype	7	22.48	3.211	25.13	<2e-16***
aliphatic glucosinolates	Experiment	1	4.16	4.156	32.53	2.44e-08***
	Residuals	363	46.38	0.128		
Long-chained	Genotype	7	0.42	0.0596	0.39	0.908
aliphatic glucosinolates	Experiment	1	0.40	0.4003	2.62	0.106
	Residuals	363	55.45	0.1528		
Aliphatic glucosinolates	Genotype	7	8.16	11.660	11.14	8.83e-13***
	Experiment	1	1.63	16.276	15.55	9.65e-05***
	Residuals	363	38.00	0.1047		
Indole glucosinolates	Genotype	7	1.56	0.2228	1.657	0.1184
	Experiment	1	0.46	0.4635	3.448	0.0641
	Residuals	363	48.80	0.1344		

*** indicates $p < 0.001$

Analyzed genotypes are wildtype or single (*hr1* , *hr4* , *hr5*), double (*hr1/4* , *hr1/5* , *hr4/5)*

or triple (*hr1/4/5*) T-DNA insertion lines.

n = 43-48 for each genotype in two independent experiments with n = 21-24 each.

<u>Short-chained aliphatic glucosinolates:</u>

3-methylthiopropyl-, 3-methylsulfinyl-, 3-hydroxypropyl-, 3-benzoyloxypropyl-, 4-methylthiobutyl-, 4-methylsulfinylbutyl-, 4-hydroxybutyl-, 4-benzoyloxybutyl-, 5-methylsulfinylpentyl-glucosinolate

<u>Long-chained aliphatic glucosinolates:</u>

7-methylthioheptyl-, 7-methylsulfinylheptyl-, 8-methylthiooctyl-, 8-methylsulfinyloctyl-glucosinolate

<u>Aliphatic glucosinolates:</u>

Short-chained aliphatic and long-chained aliphatic glucosinolates

<u>Indole glucosinolates:</u>

Indol-3-ylmethyl-, 4-methoxy-indol-3-ylmethyl-, N-methoxy-indol-3-ylmethyl-glucosinolate

Table S6: Interactors found in the BioGrid database

Gene	Name/Annotation	Interactor of
At1G14700	Purple acid phosphatase 3 (PAP3)	CYP83A1
At1G17280	Ubiquitin-conjugating enzyme 34 (UBC34)	CYP83A1
At1G21240	Wall-associated kinase 3 (WAK3)	CYP83A1
At1G29060	Golgi localized QcSNARE involved in response to salt and osmotic stress	CYP83A1
At2G26180	IQ-domain 6 (IQD6)	CYP83A1
At3G05120	GA insensitive dwarf 1A (GID1A)	CYP83B1
At3G08510	Phosphoinositide-specific phospholipase C (PI-PLC)	CYP83A1
At3G10640	VPS60.1	CYP83A1
At3G12180	Cornichon family protein	CYP83A1
At3G48890	Membrane steroid binding protein 2 (MSBP2)	CYP83A1
At4G20790	Leucine-rich repeat protein kinase family protein	CYP83A1
At4G27610	unknown	CYP83A1
At4G35950	GTP binding protein, member of ROP GTPases gene family-like (RAC6)	CYP83A1
At5G47120	Bax inhibitor 1, functions as an attenuator of cell death (BI-1)	CYP83A1
At5G52240	Membrane steroid binding protein 1 (MSBP1)	CYP83A1
At5G59650	Leucine-rich repeat protein kinase family protein	CYP83A1
